# Nutrient Level Determines Biofilm Characteristics and Subsequent Impact on Microbial Corrosion and Biocide Effectiveness

**DOI:** 10.1128/AEM.02885-19

**Published:** 2020-03-18

**Authors:** Silvia J. Salgar-Chaparro, Katerina Lepkova, Thunyaluk Pojtanabuntoeng, Adam Darwin, Laura L. Machuca

**Affiliations:** a Curtin Corrosion Centre, WA School of Mines: Minerals, Energy, and Chemical Engineering, Curtin University, Bentley, WA, Australia; b Woodside Energy Ltd., Perth, WA, Australia; Wageningen University

**Keywords:** carbon steel, microbiologically influenced corrosion, nutrient level, biofilms, microbial activity, biocide, 16S rRNA

## Abstract

The impact that nutrient level has on biofilm characteristics, biocide effectiveness, and the associated risk of microbiologically influenced corrosion (MIC) was assessed using multispecies biofilms from two different oilfield consortia. A range of microbiological, microscopy, and corrosion methods demonstrated that the continuous flow of nutrients for the microbial growth resulted in higher activity, thickness, and robustness of the biofilms formed on carbon steel, which induced greater localized corrosion compared to biofilms formed under batch, nutrient-depleted conditions. Despite of the differences in biofilm characteristics, biofilms displayed comparable susceptibilities to glutaraldehyde biocide, with similar log_10_ reductions and percent reductions of microorganisms under both nutrient conditions. Nevertheless, nutrient replenishment impacted the effectiveness of the biocide in controlling microbial populations; a higher concentration of cells survived the biocide treatment in biofilms formed under a continuous flow of nutrients. Complementary DNA-/RNA-based amplicon sequencing and bioinformatics analysis were used to discriminate the active within the total populations in biofilms established at the different nutrient conditions and allowed the identification of the microbial species that remained active despite nutrient depletion and biocide treatment. Detection of persistent active microorganisms after exposure to glutaraldehyde, regardless of biofilm structure, suggested the presence of microorganisms less susceptible to this biocide and highlighted the importance of monitoring active microbial species for the early detection of biocide resistance in oil production facilities.

**IMPORTANCE** Microbiologically influenced corrosion (MIC) is a complex process that generates economic losses to the industry every year. Corrosion must be managed to prevent a loss of containment of produced fluids to the external environment. MIC management includes the identification of assets with higher MIC risk, which could be influenced by nutrient levels in the system. Assessing biofilms under different nutrient conditions is essential for understanding the impact of flow regime on microbial communities and the subsequent impact on microbial corrosion and on the effectiveness of biocide treatment. This investigation simulates closely oil production systems, which contain piping sections exposed to continuous flow and sections that remain stagnant for long periods. Therefore, the results reported here are useful for MIC management and prevention. Moreover, the complementary methodological approach applied in this investigation highlighted the importance of implementing RNA-based methods for better identification of active microorganisms that survive stress conditions in oil systems.

## Abstract (precis)

Microbiologically influenced corrosion (MIC) is a complex process that generates economic losses to the industry every year. Corrosion must be managed to prevent a loss of containment of produced fluids to the external environment. MIC management includes the identification of assets with higher MIC risk, which could be influenced by nutrient levels in the system. Assessing biofilms under different nutrient conditions is essential for understanding the impact of flow regime on microbial communities and the subsequent impact on microbial corrosion and on the effectiveness of biocide treatment. This investigation simulates closely oil production systems, which contain piping sections exposed to continuous flow and sections that remain stagnant for long periods. Therefore, the results reported here are useful for MIC management and prevention. Moreover, the complementary methodological approach applied in this investigation highlighted the importance of implementing RNA-based methods for better identification of active microorganisms that survive stress conditions in oil systems.

## INTRODUCTION

Microorganisms have a natural tendency to attach to wet surfaces producing extracellular polymeric substances (EPS) that initiate or enhance the attachment process. Surface colonization is followed by the formation of biofilm structures that provide several ecological advantages to the microorganisms over planktonic growth (1). Sessile microorganisms have greater access to nutrients and other resources accumulating at surfaces, superior environmental stability, enhanced opportunities for interactions such as horizontal gene transfer and cometabolism, and protection from physical disturbances and antimicrobial compounds (2). Microorganisms can attach to different surfaces, organic and inorganic, and in distinct environmental conditions. Hence, in industrial assets, biofilms have been found on a wide range of metals and alloys, including carbon steel (3), stainless steel (4), aluminum (5), and copper (6), among others. The metabolic activities of the microorganisms attached to the metallic surfaces can alter the physical and chemical properties of these materials, changing their susceptibility to corrosion (7). This process where microorganisms modify the kinetics of corrosion reactions is known as microbiologically influenced corrosion (MIC) (8). Microorganisms can initiate, accelerate, or facilitate corrosion reactions by several mechanisms, including the formation of concentration cells, the production of corrosive metabolites, the dissolution of protective layers, the uptake of electrons directly from the metal, and the production of layers that are not protective (9). Most MIC is localized in nature occurring at the substratum-electrolyte interphase, where deep penetration into the base metal is observed (10). This phenomenon generates economic losses principally to the oil and gas industry, which have been estimated in billions of dollars per year in the United States alone (11). In addition to material deterioration, microorganisms forming biofilms may cause other issues, such as biological fouling, heat transfer loss, and a decrease in product quality in several industries.

Biofilms on metallic surfaces are mainly composed of microbial cells that are packed with EPS, corrosion products, nucleic acids, traces of inorganic minerals, and some organics adsorbed from the bulk fluid. Biofilms are heterogeneous with microcolonies of cells separated by interstitial channels, voids, and pores that allow diffusion and transport of energy sources and waste products (12, 13). This heterogeneity influences biofilm activity by its effect on the rates of nutrient transport and consumption (14). Likewise, the structure and morphology of biofilms can vary with the growth rate of microorganisms and the types and levels of energy sources available (13). The physiology of sessile cells is particularly complex and is markedly different from those growing free in the bulk fluid (planktonic). Moreover, the physiological status of the microorganisms conforming the biofilm is diverse and is determined by the location of the cells within the multiple layers of the biofilm (15). It has been proposed that microorganisms located in the upper regions of the biofilm are more active due to greater accessibility to nutrients and greater ease of discharging of metabolic waste products compared to cells at the bottom of the biofilm (15).

In oil production facilities, microorganisms find sufficient essential nutrients and carbon sources, including petroleum hydrocarbons, volatile fatty acids, and other fermentation products distributed across the production system through produced water (16). Nonetheless, design features and operational conditions in these facilities may create zones with different flow regimes, such as dead legs, that limit the availability of such nutrients (17). Higher flow velocity can provide an increased supply of nutrients for biofilms formed on metal surfaces, but it may also result in biofilm removal (13, 18). In contrast, the sections of equipment and piping that remain stagnant for long periods during operation allow microorganisms to settle but also provide less flow of nutrients. Environments with an abundant supply of nutrients would be expected to favor attachment and biofilm formation; however, previous studies have shown a greater tendency to form biofilms under low-nutrient or starvation conditions (18). Likewise, in cases of electrical MIC, microorganisms have been shown to switch from soluble electron donors to metallic iron as the only energy source to carry out microbial activities under starving conditions (19). This mechanism has been shown to lead to higher corrosion rates in the laboratory (20). However, under these conditions biofilms are known to operate under stress and are unable to multiply because metal does not provide the carbon that cells need for growth (20). Although under starvation cells can scavenge nutrients from the EPS and dead cells, reductions in cell counts are frequently observed when carbon sources are depleted (19, 21). In the field, MIC has been mainly associated with low velocity or infrequently flooded systems, dead legs and pipelines with solid deposit accumulation that cannot be adequately cleaned. The increased MIC risk in these areas has been related to the more favorable conditions for microbial settlement and biofilm formation (22); nonetheless, the availability of nutrients is expected to play a significant role in biofilm activity and the resulting MIC rates. However, the relationship between nutrient availability and the risk of MIC remains undefined for stagnant areas. Since the majority of MIC found in oilfields is attributed to corrosive metabolites (23), it is implied that the presence of nutrients in the system that allow microorganisms to carry out metabolic activities and proliferate is critical for catalytic MIC to occur.

In addition to the effect of nutrients on MIC, it is also unclear whether the biofilm physiological status plays a role in the effectiveness of biocide treatments used to prevent MIC. It has been reported that the restriction of essential nutrients to microorganisms can markedly affect their susceptibility to antimicrobial substances (24, 25). Several researchers have argued that actively growing cells are more susceptible to antibiotics than those cells that are slowly or not actively growing. The greater antimicrobial resistance in such cells within the biofilm has been attributed to the less-permeable membranes present in slow-growing cells (15). Moreover, biofilm structure can also play an important role in biocide efficacy by affecting the diffusion of antimicrobial compounds through biofilm layers and reducing the concentration of antimicrobial compounds reaching the cells at the bottom of biofilms. Therefore, these cells will be exposed to sublethal concentrations of biocidal compounds and will have sufficient time to switch on the expression of antimicrobial-resistant factors and antimicrobial-degrading enzymes (26). Hence, it is important to identify the environmental stresses that are likely to influence the physiology of the microorganisms in industrial systems to develop better approaches and practices to prevent the pervasive and detrimental corrosion caused by biofilms.

This investigation was conducted to identify changes in biofilms in response to nutrient level and to examine how these changes influence the severity of MIC and the effectiveness of glutaraldehyde, a chemical biocide commonly used to prevent MIC in the industry. Considering that natural and industrial environments are colonized by multispecies consortia and that multispecies biofilms have been poorly studied so far, this study was conducted using multispecies biofilms from microbial consortia recovered from oilfields. Two microbial consortia from different oilfields were exposed to the same conditions separately to determine whether different microbial communities will display similar response to the nutrient regime. The variation in the microbial composition of biofilms on carbon steel was studied by complementary DNA-/RNA-based amplicon sequencing from the 16S rRNA gene and transcripts, as used elsewhere (27). The physiological status of the microbial communities was assessed by the estimation of the adenylate energy charge (AEC), which provided information about the metabolic state of the cells before and after biocide evaluation. Biofilm morphology and thickness were studied via visible-light, confocal, and scanning electron microscopy analyses, and the results were correlated with corrosion rates. Dissimilarities found in the biofilms developed on metals exposed to different nutrient regimes provided valuable information about the variation expected in microbial communities throughout oil production facilities, which should be accounted for when assessing the risk of MIC and for establishing MIC mitigation and monitoring strategies.

## RESULTS

### Effect of nutrient replenishment on biofilm characteristics. (i) Biofilm microscopy analysis.

**FIG 1:**
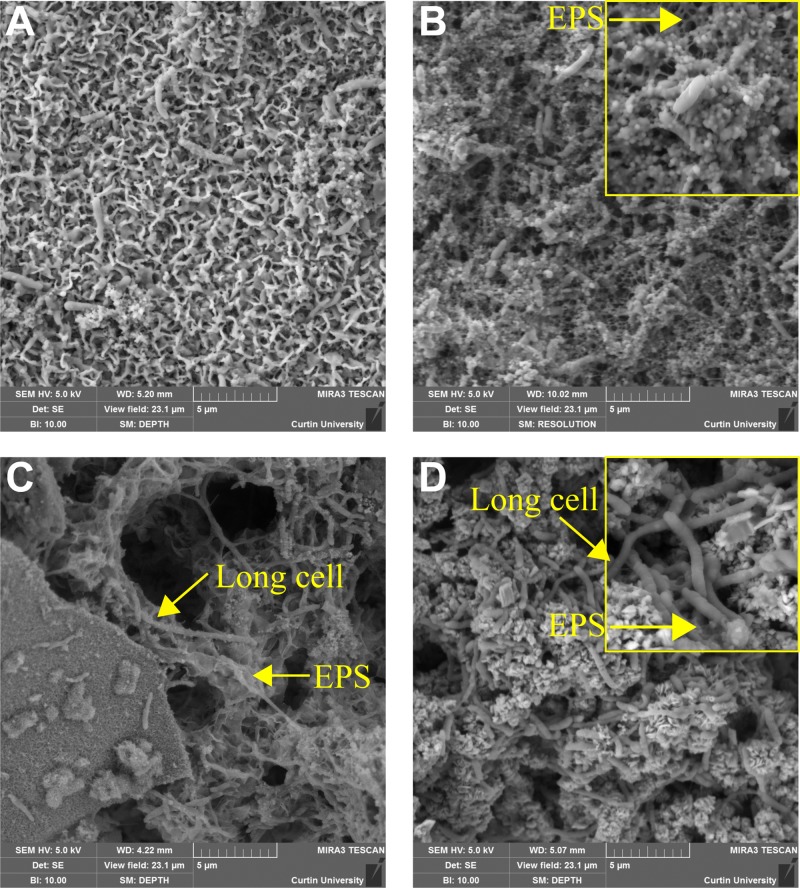
FESEM views of the biofilms/corrosion products formed over carbon steel coupons at different nutrient conditions. (A) Consortium 1 under batch conditions; (B) consortium 1 with continuous replenishment; (C) consortium 2 under batch conditions; (D) consortium 2 with continuous replenishment.

Field emission scanning electron microscopy (FESEM) morphological views of the biofilms formed by both microbial consortia over carbon steel samples exposed to different nutrient conditions are shown in Fig. 1. Biofilms grown under batch conditions contained fewer cells (Fig. 1A and C) than biofilms grown under a continuous flow of nutrients (Fig. 1B and D). The same result was obtained for the two microbial consortia evaluated. A particular pattern in biofilm distribution was observed in microbial consortium 1. Cells exposed to the batch conditions were embedded in a thin layer of corrosion products; no EPS-like structures were evident on the surface (Fig. 1A). In contrast, biofilms formed by the same consortium under continuous nutrient replenishment exhibited microbial cells interconnected by organic structures compatible with pili filaments or EPS (Fig. 1B). Biofilms formed by microbial consortium 2 were denser in corrosion products and cells than the biofilm of consortium 1 at each nutrient condition. Different from consortium 1, cells in the biofilms formed under batch conditions for consortium 2 were embedded on EPS-like structures (Fig. 1C), which were also evident in the biofilms under continuous replenishment. As expected, dissimilarities in the morphology of microbial cells were also evidenced using FESEM analysis. The presence of long rod cells was detected in microbial consortium 2 (Fig. 1C and D), a morphology that was not visualized in microbial consortium 1, which indicated differences in microbial composition between consortia.

**FIG 2:**
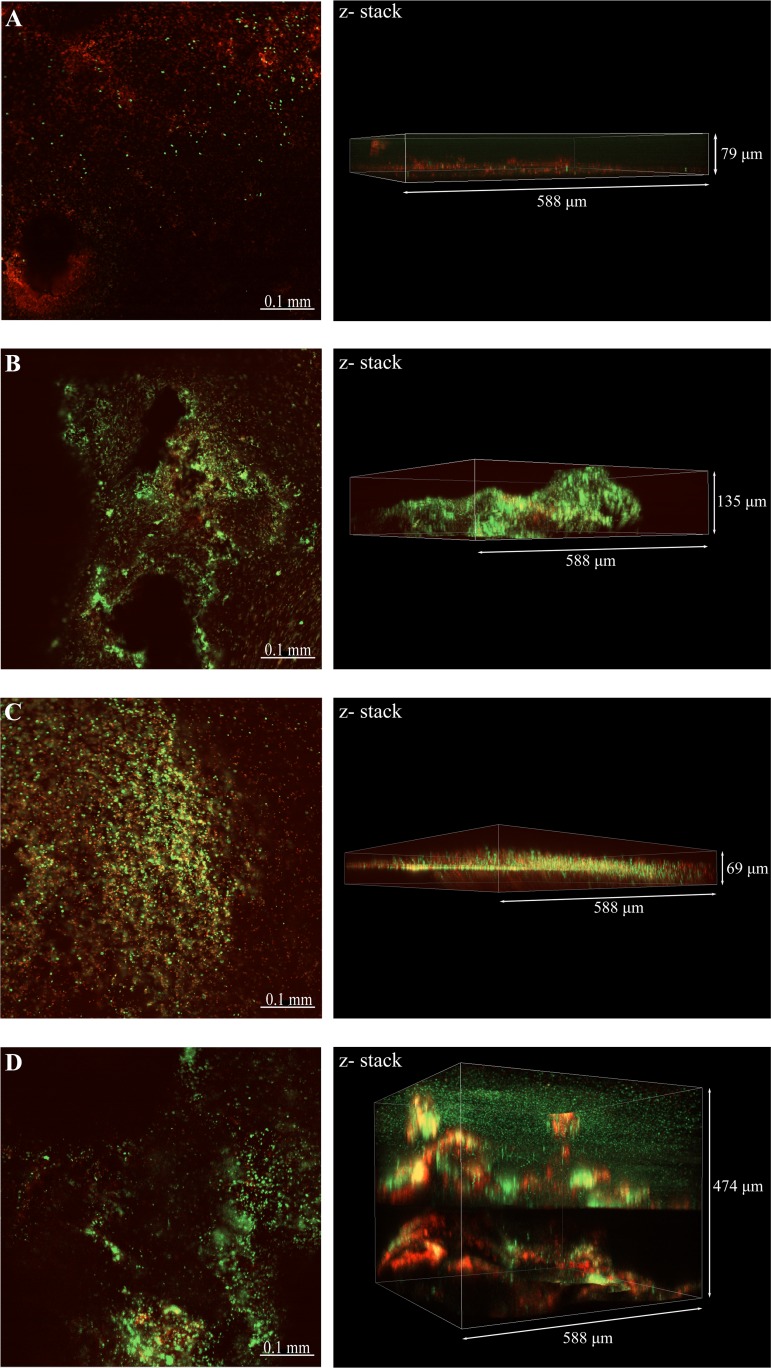
CLSM images of biofilms formed over carbon steel coupons at different nutrient conditions. (A) Consortium 1 under batch conditions; (B) consortium 1 with continuous replenishment; (C) consortium 2 under batch conditions; (D) consortium 2 with continuous replenishment. Live cells, green stain; dead cells, red stain.

Figure 2 shows the confocal laser scanning microscopy (CLSM) images of the biofilms with differentiation of live and dead cells by the fluorescent dye. This analysis revealed differences in the structure and distribution of live and dead cells in the biofilms as a result of nutrient replenishment. As observed in the FESEM analysis, denser biofilms were observed on steel coupons exposed to a continuous replenishment of nutrients for both consortia (Fig. 2B and D); z-stack images indicated that thicker biofilms were formed under these conditions than under batch conditions (Fig. 2A and C). Likewise, live and dead staining indicated that biofilms grown under batch conditions had a higher density of dead cells, a difference that was more marked in biofilms formed by microbial consortium 1. It was also noticed that, for both consortia, thicker biofilms formed under continuous replenishment of nutrients had a higher concentration of dead cells at the bottom layers of the biofilm than at the top layers.

**FIG 3:**
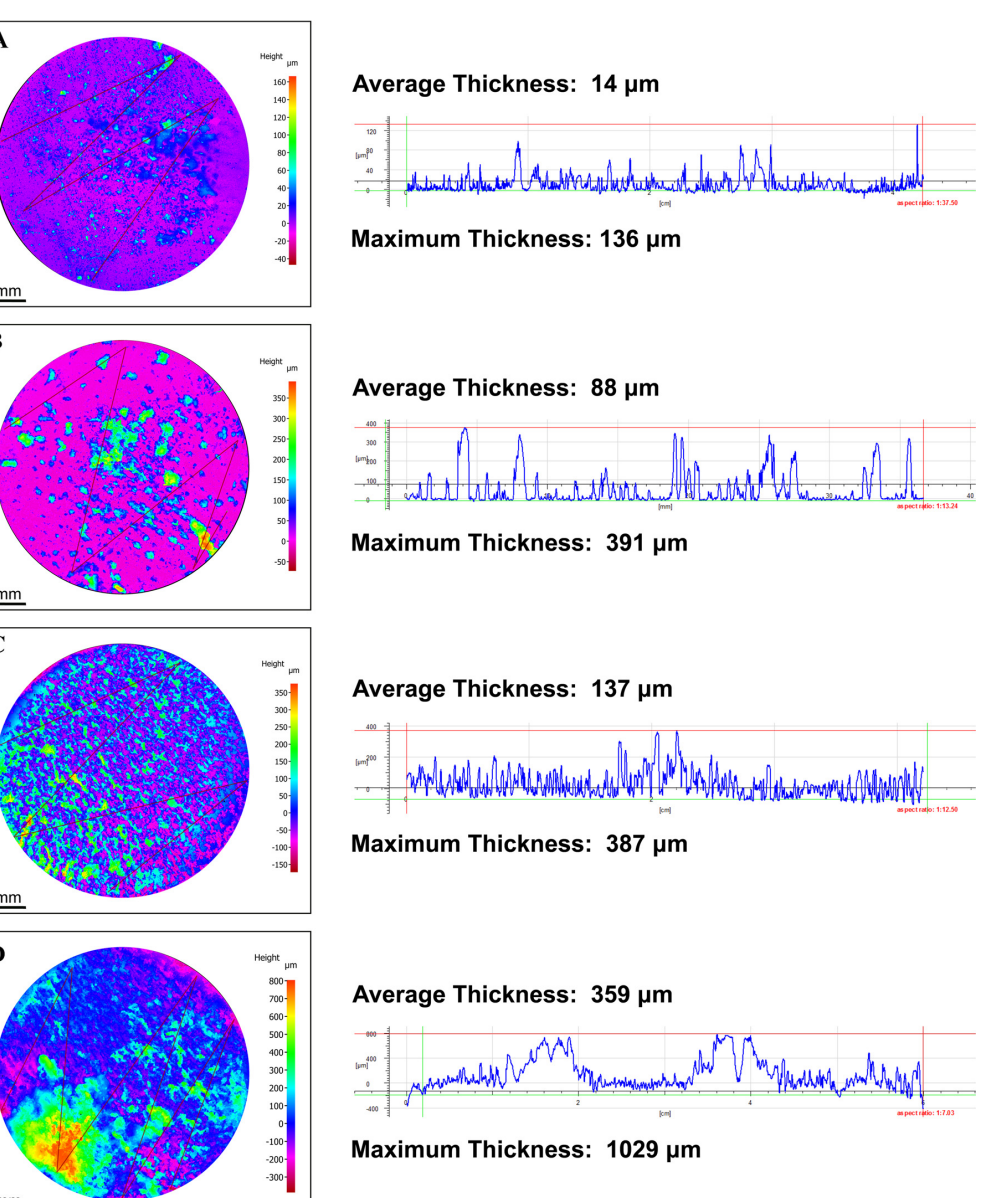
3D reconstruction of the corrosion products and biofilm profiles formed over the surface of carbon steel coupons under different nutrient conditions. (A) Consortium 1 under batch conditions; (B) consortium 1 with continuous replenishment; (C) consortium 2 under batch conditions; (D) consortium 2 with continuous replenishment.

The heterogeneous distribution of the biofilms over the metal surface did not allow us to conduct measurements of the maximum biofilm thickness using CLSM analysis. Therefore, the thickness of wet biofilms was not determined. Three-dimensional (3D) reconstruction of the corrosion products and biofilm profiles (dried samples) was performed with a 3D profilometer microscope; the images are shown in Fig. 3. This analysis revealed differences in the distribution of the corrosion products and biofilms formed over the metal surface by each microbial consortium. The results of the 3D analysis confirmed that consortium 2 created thicker biofilms that, together with corrosion products, covered the complete surface of the steel samples (Fig. 3C and D). In contrast, consortium 1 formed thinner biofilms that were irregularly distributed over the metal surface (Fig. 3A and B). Differences in biofilm thickness formed by the same consortia but under different nutrient conditions were also confirmed via 3D profiling; thicker biofilms were seen in reactors with a continuous replenishment of nutrients (Fig. 3B and D). The same pattern was observed for the two microbial consortia evaluated.

### (ii) Microbial concentration.

Most probable numbers (MPNs) of biofilm microbial communities revealed that the continuous replenishment of nutrients stimulated biomass production and biofilm growth, with 1 to 3 orders of magnitude more cells compared to batch conditions (Table 1). Similar results were obtained for the two microbial consortia evaluated. The concentration of sulfide-producing prokaryotes (SPP) was significantly higher in the biofilms formed by consortium 2 under nutrient replenishment, with 4 orders of magnitude more cells than the biofilm formed by consortium 1 under the same conditions. Microorganisms in consortium 2 were recovered from the floating production storage and offloading (FPSO) with high sulfate concentration in the produced water, which could explain the greater numbers of microorganisms with the capability of using sulfate for energy acquisition. Enumeration of planktonic cells showed that the concentration of SPP in the continuous reactors was lower than in the batch reactors (see Table S4 in the supplemental material), which could be related to the contribution of nutrients to the synergistic or antagonistic interactions among species within biofilms. The accurate concentration of anaerobic cells in planktonic communities could not be determined since the highest serial dilution vials showed positive growth.

**TABLE 1:** Most probable numbers of viable cells in biofilm communities exposed to different nutrient conditions^*a*^

Consortium	Condition	MPN SPP (cells/cm^2^)	95% confidence limit		MPN ANA (cells/cm^2^)	95% confidence limit	
			Lower	Higher		Lower	Higher
1	Batch	1.50E+05	3.70E+04	6.30E+07	7.49E+09	1.70E+09	1.50E+12
	Continuous	4.60E+06	9.00E+05	9.20E+09	2.10E+11	4.00E+10	9.03E+13
2	Batch	2.40E+05	4.20E+04	2.40E+08	1.50E+09	3.70E+08	6.30E+11
	Continuous	1.10E+10	1.80E+09	4.51E+13	>1.1E+12	4.20E+11	

a Most probable numbers (MPN) were determined from three-tube serial dilutions. SPP, sulfide-producing prokaryotes; ANA, total anaerobic microorganisms.

### (iii) Microbial activity.

ATP, ADP, and AMP concentrations and the corresponding energy charge measured in the biofilm communities are presented in Table 2. The results indicate that biofilms grown under batch conditions had lower concentrations of ATP, ADP, and AMP than biofilms exposed to continuous replenishment of nutrients, which was correlated with the lower biomass found under batch conditions. Statistical analysis revealed that differences in the adenosine nucleotide concentrations between batch and continuous replenishment biofilms were significant (*P* ≤ 0.05; see Table S5 in the supplemental material). Similar results were obtained for both microbial consortia evaluated. Analysis of the physiological status of the biofilms indicated that microbial communities were under stress conditions (Table 2); all of the microbial populations had an AEC below 0.75. Greater stress was observed in the biofilm formed under batch conditions by consortium 1 with AEC below 0.5. Differences in the relative proportion of the three nucleotides were observed in the biofilms as a result of nutrient availability. Higher AMP proportions were measured in the biofilms formed by both consortia under batch conditions, suggesting a major proportion of dormant cells under this condition. Differences in the ATP proportion between batch and continuous replenishment were not consistent in both consortia. Consortium 1 showed a lower ATP proportion under batch conditions than under continuous replenishment, whereas consortium 2 showed the opposite pattern. It should be noted that even though consortium 2 exhibited a higher ATP proportion when exposed to batch conditions than when exposed to continuous replenishment conditions, the total proportion of ATP and ADP in the continuous reactor was higher than that in the batch reactor, indicating more stored energy in reactors with replenishment of nutrients, as seen for consortium 1.

**TABLE 2:** Average adenosine nucleotide concentrations and adenylate energy charges of the biofilm communities grown under different nutrient conditions

Parameter	Avg ± SD^*a*^			
	Consortium 1		Consortium 2	
	Batch	Continuous	Batch	Continuous
ATP (pg/cm^2^)	765 ± 520	21,523 ± 19,098	634 ± 359	81,594 ± 38,026
ADP (pg/cm^2^)	896 ± 651	17,362 ± 10,781	157 ± 128	82,181 ± 28,115
AMP (pg/cm^2^)	1,269 ± 925	4,057 ± 289	357 ± 175	33,520 ± 3,526
Total adenylates (ng/cm^2^)	2.9 ± 2.1	42.9 ± 3.0	1.1 ± 0.6	197.3 ± 69
AEC	0.49 ± 0.13	0.67 ± 0.08	0.61 ± 0.21	0.61 ± 0.05
% ATP	26 ± 11.3	50 ± 9.9	54 ± 27.8	40 ± 5.1
% ADP	31 ± 0.7	40 ± 3.9	15 ± 14.0	42 ± 1.4
% AMP	43 ± 13.5	10 ± 6.0	31 ± 14.9	18 ± 4.2

a Errors represent standard deviations from three independent replicates.

The concentration of the adenosine nucleotides in planktonic cells showed a pattern similar to that observed in sessile populations; higher concentrations of ATP, ADP, and AMP were observed in cells exposed to a continuous replenishment of nutrients (see Table S6 in the supplemental material). The AEC values of planktonic cells in batch reactors of both consortia was below 0.5, which indicated that microorganisms were under severe stress. In contrast, planktonic cells in reactors with continuous replenishment had higher AEC; however, as detected in the biofilm communities, AEC values were still below 0.75. It was noticed that planktonic communities had a higher concentration of adenosine nucleotides than biofilm communities in both consortia, indicating higher cell numbers in an active state in the test solution compared to microbial cells attached to the metal surface.

### (iv) Biofilm community composition.

**FIG 4:**
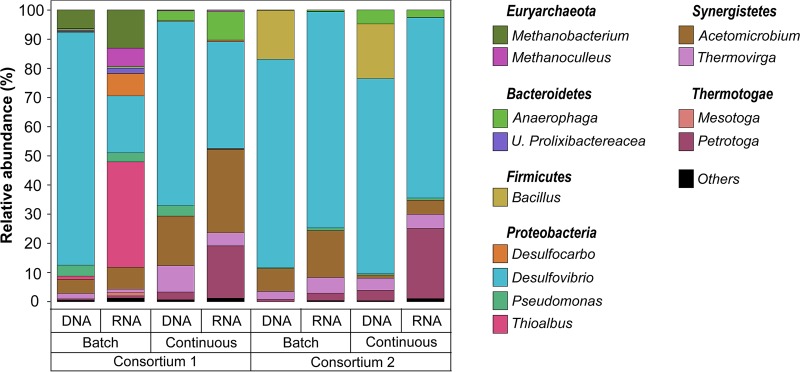
Total (DNA-based) and active (RNA-based) microbial community composition of biofilms exposed to different nutrient conditions. The results show the mean relative abundances of microbial communities classified at the genus level from 16S rRNA sequencing (*n* = 2). Genera with relative abundances lower than 1% in all samples were summarized in the artificial group “Others.”

Total and active microorganisms in biofilms grown under different nutrient conditions were identified through DNA and RNA-based amplicon sequencing. A total of 1,705,081 high-quality sequences were obtained after bioinformatics processing of the raw reads. These sequences were taxonomically classified into 29 microbial genera. Only the genera with relative abundances of ≥1% are presented in Fig. 4. The complete list of biofilm microbial composition is presented in Table S7 in the supplemental material. Molecular identification of the microorganisms showed that both biofilms exhibited similar microbial populations, with variations related to the presence of methanogenic species in consortium 1 that were not detected in the consortium 2. Differences in the relative abundances of dominant genera and in the composition of the low-abundance microorganisms were also noted. The results of the alpha diversity analysis indicated that biofilms grown under continuous replenishment of nutrients were more diverse (Table S8), suggesting a more complex community when a higher level of nutrients was available in the reactor.

**FIG 5:**
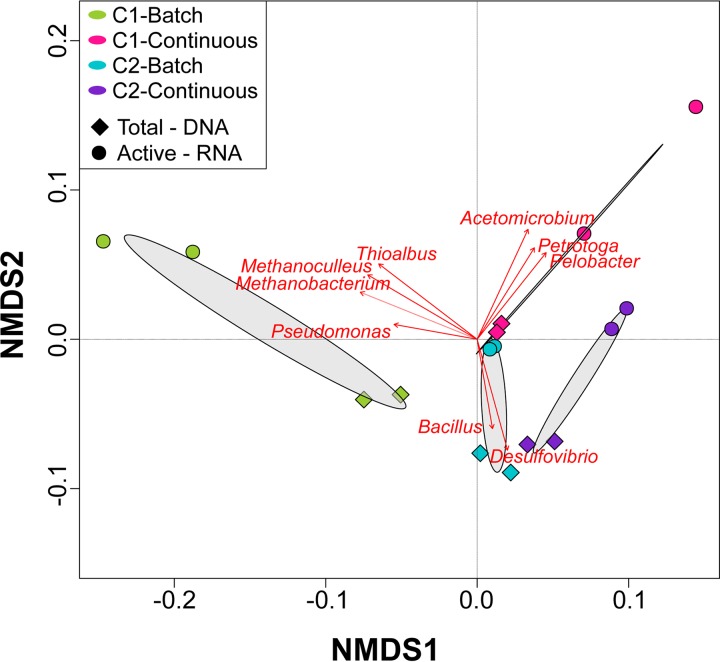
NMDS of total and active microbial communities in biofilms exposed to different nutrient conditions. Dominant microorganisms that were significantly correlated (*P* ≤ 0.05) to microbial community structure are indicated by arrows.

Comparison of the DNA and RNA profiles showed differences in the relative abundances of the total and active populations in each consortium under the conditions evaluated, which were also reflected in the NMDS ordination analysis (Fig. 5). Total communities in microbial consortium 1 were dominated by *Desulfovibrio* spp. (80 and 63%, batch and continuous mode, respectively), whereas, in the active communities this genus had a lower relative abundance (20 and 37%, batch and continuous mode, respectively). Other genera showed the opposite pattern, i.e., lower abundances in the total community and higher abundances in the active community. Substantial differences in the biofilm microbial composition as a result of nutrients were observed in consortium 1. For this consortium, the genera *Thioalbus*, *Methanobacterium*, and *Methanoculleus* were only detected abundantly in the communities formed under batch conditions. These microorganisms, together with *Desulfovibrio*, *Acetomicrobium*, and *Desulfocarbo*, were the most dominant genus in the active community. In contrast, the active biofilm community formed under continuous mode was dominated by *Desulfovibrio*, *Acetomicrobium*, *Petrotoga*, and *Anaerophaga*. Total and active biofilm communities in consortium 2 under both conditions were equally dominated by *Desulfovibrio*. The most considerable difference between total and active communities was observed in the relative abundances of the genera *Bacillus*, *Acetomicrobium*, and *Petrotoga*. The last two showed a higher abundance in the active communities, a pattern similar to that observed in microbial consortium 1. In contrast, *Bacillus* that was one of the dominant populations in the total communities of both conditions was not detected abundantly in the active communities. The main difference in the biofilm composition of consortium 2 as a result of the nutrient level was associated with the increase in the relative abundance of the genera *Petrotoga* and *Anaerophaga* in the continuously replenished reactor, a pattern similar to that observed in microbial consortium 1.

### Effect of nutrient replenishment on microbial corrosion. (i) Corrosion measurements.

**FIG 6:**
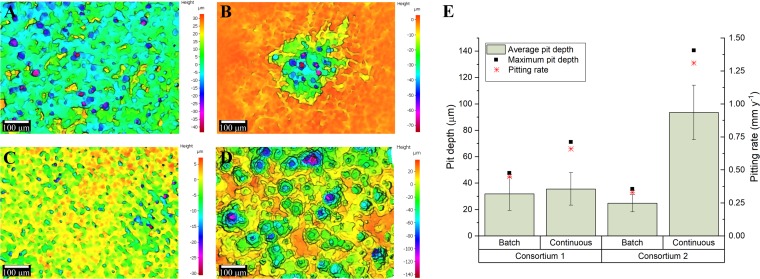
Localized corrosion analysis of carbon steel coupons exposed to different nutrient conditions. (A to D) 3D optical microscope surface images of carbon steel coupons. (A) Consortium 1 under batch conditions; (B) consortium 1 with continuous replenishment; (C) consortium 2 under batch conditions; (D) consortium 2 with continuous replenishment. (E) Average pit depths and pitting rates calculated from the maximum pit depth. Error bars represent standard deviations of the 10 deepest pits found in three independent replicates.

Surface profilometry analysis revealed that biofilms from the two different consortia triggered localized corrosion under both nutrient conditions (Fig. 6). The average pit depth (calculated from the ten deepest pits found in the three coupons analyzed from each reactor), the maximum pit depths, and the pitting rates are shown in Fig. 6E . For the two consortia, deeper pits were found in reactors with a continuous replenishment of nutrients than in reactors under batch conditions; however, the difference in the pit depth according to the nutrient level was only significant for consortium 2 (*P* ≤ 0.05; Table S5). The extent of MIC was different for each consortium; under batch conditions consortium 1 caused higher pitting rates than did consortium 2, whereas under a continuous replenishment of nutrients consortium 2 resulted in more localized corrosion compared to consortium 1. The abiotic reactor did not show evidence of localized corrosion damage (pits < 10 μm) (see Fig. S1 in the supplemental material), which indicated that the surface deterioration observed in the metal samples was the result of microbial activity. The average corrosion rates calculated from the coupon’s mass loss are presented in Table S9. The results showed a different trend in both consortia. Microorganisms of consortium 2 induced higher general corrosion rates when continuous replenishment of nutrients was established. In contrast, microorganisms of consortium 1 triggered similar general corrosion rates independently of nutrient levels.

### (ii) Cross-sectional analysis of corrosion products.

**FIG 7:**
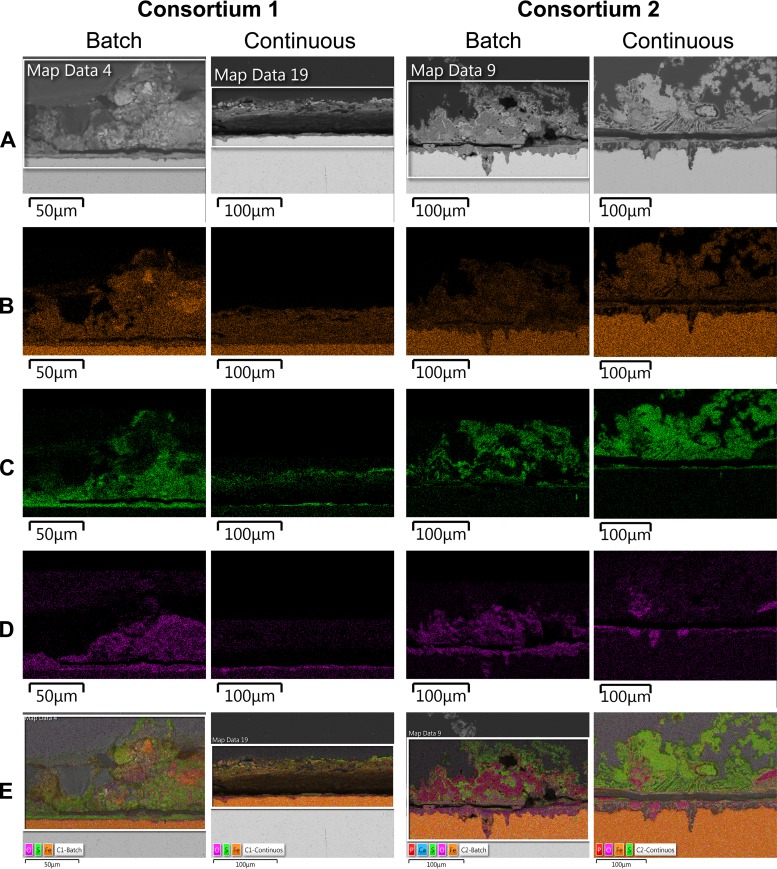
EDS-elemental mapping of cross-sectioned coupons exposed to different nutrient conditions. (A) SEM image; (B) iron map; (C) sulfur map, (D) oxygen map; (E) combined elemental map.

Cross-sectional images of corrosion products and biofilms attached to the metal samples also showed differences in distribution and thickness of biofilms in response to nutrient conditions (Fig. S2). Likewise, corroded areas under the biofilms were examined by cross-sectional analysis. It was seen that coupons exposed to the microbial consortium 2 exhibited greater corroded areas than coupons exposed to consortium 1 under both nutrient conditions, which was supported by the higher mass loss evidenced in the coupons exposed to consortium 2 (Table S9). The elemental maps of the cross-sectional surface analysis are shown in Fig. 7. The major elements detected in coupons exposed to all conditions were iron, sulfur, and oxygen. In the coupons exposed to microbial consortium 2, phosphorous and calcium were also detected (Fig. S3). Corroded areas of all coupons were mainly covered by Fe and O, whereas the top layers were mainly composed of Fe and S. Layered images of coupons exposed to microbial consortium 2 showed a higher concentration of phosphorous in the top layers of the coupon exposed to batch conditions compared to coupons exposed to the continuous flow of nutrients; this element was mapped in same areas with oxygen. A carbon distribution map could not be obtained due to the carbon-based nature of the epoxy resin used for mounting of the samples.

### Effect of nutrient replenishment on biocide effectiveness. (i) Microbial concentration.

The microbial concentrations of SPP and other anaerobic microorganisms in the biofilms after the biocide treatment are presented in Table 3. The results show that glutaraldehyde treatment reduced considerably the concentration of viable microorganisms in biofilms before the treatment, killing 99.99% of the initial population with maximum log_10_ reduction of 8 orders under all conditions (Table 4). Similar results were observed for both consortia and both nutrient conditions. However, differences in the number of viable cells remaining after biocide treatment were observed based on nutrient conditions. Biofilms formed in batch reactors showed a lower number of viable residual cells than biofilms formed under continuous nutrient replenishment (Table 3), which indicated that higher biocide effectiveness in controlling the biofilm populations was achieved under nutrient-depleted conditions. The same results were obtained from both microbial consortia.

**TABLE 3:** Most probable numbers of viable cells for biofilm communities after biocide treatment^*a*^

Consortium	Condition	MPN SPP (cells/cm^2^)	95% confidence limit		MPN ANA (cells/cm^2^)	95% confidence limit	
			Lower	Higher		Lower	Higher
1	Batch	3.60E+00	1.70E−01	6.48E+01	3.94E+01	9.00E+00	7.74E+03
	Continuous	2.80E+02	8.70E+01	2.63E+04	1.22E+03	3.70E+02	6.30E+05
2	Batch	< 3		9.50E+00	7.40E+00	1.30E+00	1.48E+02
	Continuous	2.80E+02	8.70E+01	2.63E+04	4.32E+03	9.00E+02	9.20E+06

a The MPNs were determined from three-tube serial dilutions. SPP, sulfide-producing prokaryotes; ANA, total anaerobic microorganisms.

**TABLE 4:** Log_10_ reductions and percent reductions for biofilm communities after biocide treatment^*a*^

Consortium	Condition	Log_10_ reduction		% reduction	
		SPP	ANA	SPP	ANA
1	Batch	4.62	8.24	99.99	99.99
	Continuous	4.22	8.15	99.99	99.99
2	Batch	5.38	8.31	99.99	99.99
	Continuous	7.59	8.48	99.99	99.99

a SPP, sulfide-producing prokaryotes; ANA, total anaerobic microorganisms.

### (ii) Microbial activity.

The ATP concentration in all biofilms was reduced after exposure to glutaraldehyde biocide (Table 5). A higher percentage of reduction in ATP levels was observed in biofilms grown under continuous replenishment of nutrients (72 and 84%, consortia 1 and 2, respectively) than in those under batch conditions (27 and 29%, consortia 1 and 2, respectively). As expected, the total concentration of adenosine nucleotides was reduced with the biocide treatment, which was likely related to the release of these molecules during the cell membrane lysis of the microorganisms killed by the treatment. Measurements of the AEC in biofilms after biocide treatment were below 0.5 in all communities, indicating that on average, microorganisms that remained viable in biofilms were senescent or severely stressed by the presence of the biocide. Similar results were observed for both nutrient conditions, although it was noticed that the AEC of the biofilm of consortium 1 formed under batch conditions, which was already under severe stress before the treatment, was not considerably changed by biocide treatment. It was seen that biocide exposure caused an increase in the proportion of AMP in the biofilms formed under nutrient replenishment, whereas this proportion was reduced in the biofilms formed under batch, nutrient-depleted conditions; same results were observed for both consortia. Changes in the AMP proportion are associated with variation in dormancy and/or stress-responsive processes within microbial populations; therefore, the disparity observed in the variation on the proportion of this molecule between nutrient conditions suggested an impact of the nutrient availability in the population affected by the biocide.

**TABLE 5:** Average adenosine nucleotide concentrations and adenylate energy charges of biofilm communities after biocide treatment

Parameter	Avg ± SD^*a*^			
	Consortium 1		Consortium 2	
	Batch	Continuous	Batch	Continuous
ATP (pg/cm^2^)	560 ± 109	3,425 ± 472	451 ± 54	22,521 ± 2,008
ADP (pg/cm^2^)	1,132 ± 513	10,500 ± 1,484	1,285 ± 132	64,410 ± 9,353
AMP (pg/cm^2^)	726 ± 457	3,654 ± 714	644 ± 109	48,978 ± 5,221
Total adenylates (ng/cm^2^)	2.4 ± 1.0	17.6 ± 2.6	2.4 ± 0.1	135.9 ± 16.5
AEC	0.48 ± 0.05	0.49 ± 0.01	0.46 ± 0.03	0.49 ± 0.003
% ATP	24 ± 1.5	19 ± 0.4	19 ± 1.9	17 ± 0.7
% ADP	47 ± 0.4	60 ± 1.3	54 ± 2.9	47 ± 1.2
% AMP	29 ± 1.1	21 ± 1.1	27 ± 4.7	36 ± 0.6

a Errors represent standard deviations from three independent replicates.

### (iii) Biofilm community composition.

**FIG 8:**
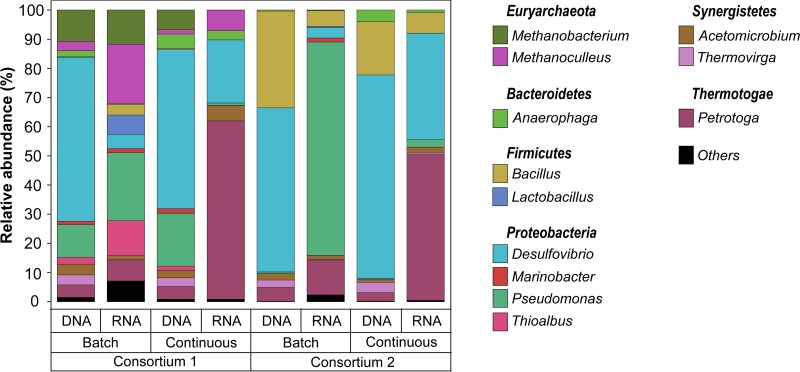
Total (DNA-based) and active (RNA-based) microbial community composition of biofilms after biocide treatment. The results show the mean relative abundances of microbial communities classified at the genus level from 16S rRNA sequencing (*n* = 2). Genera with relative abundances lower than 1% in all samples were summarized in the artificial group “Others.”

**FIG 9:**
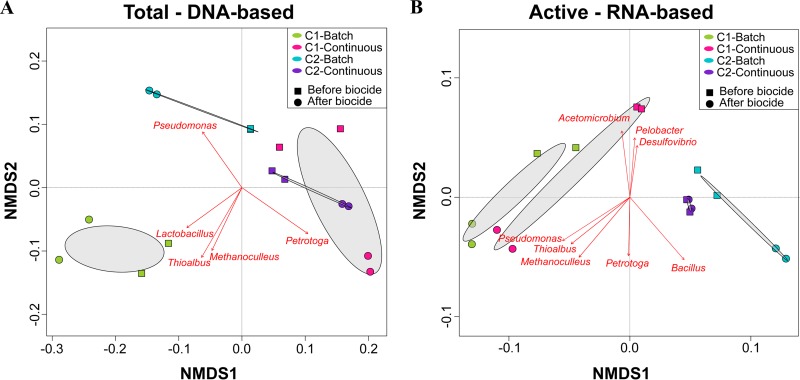
NMDS of total (A) and active (B) microbial communities after biocide treatment. Dominant microorganisms that were significantly correlated (*P* ≤ 0.05) to microbial community structure are indicated by arrows.

A total of 1,351,660 high-quality sequences were obtained after bioinformatics processing of the raw reads from the DNA and RNA-based amplicon sequencing of biofilms after biocide. These sequences were taxonomically classified into 52 microbial genera. Only the genera with relative abundances of ≥1% are presented in Fig. 8. Molecular identification of the microorganisms showed that biocide shifted the relative abundances of the predominant populations in the biofilm communities, causing changes in the community structure (Fig. 9). More microbial genera were detected in the biofilm communities after biocide treatment, which might be related to the reduction in the cell concentration of dominant species susceptible to the treatment, allowing the detection of more species with lower abundance (rare taxa) in the biofilm. The proportion of the genera *Desulfovibrio*, *Acetomicrobium*, *Thioalbus*, and *Thermovirga* in the total and active communities was reduced after glutaraldehyde exposure, indicating susceptibility of these populations to the chemical. An opposite pattern was observed for the genera *Petrotoga*, *Methanobacterium*, *Methanoculleus*, *Pseudomonas*, and *Bacillus*, which showed higher relative abundances, especially in the active communities after biocide treatment. These findings suggested that these populations were less susceptible to biocide treatment. It is important to clarify that a higher relative abundance after chemical treatment does not mean proliferation or growth of these populations during biocide exposure; increases are likely related to changes in the proportions of dominant microorganisms in the remaining viable cells due to the lysis of other dominant species that were more susceptible to the treatment. In addition, microbial composition analysis revealed that the reduction in the relative abundance of the *Desulfovibrio* genus in the active communities was greater in biofilms formed under batch conditions than in biofilms formed in the nutrient replenishment scenario. Likewise, it was noticed that the increase in the relative abundance of *Pseudomonas* and *Petrotoga* in the active community after biocide injection was associated with nutrient conditions. *Pseudomonas* abundance increased more significantly in biofilms formed in batch reactors, whereas *Petrotoga* showed higher abundance in biofilms formed under continuous nutrient replenishment. These findings may indicate that nutrient replenishment could have played a role in the survival of these microorganisms despite biocide treatment.

## DISCUSSION

We evaluated here the impact that nutrients have on biofilm characteristics and the subsequent impact on carbon steel corrosion and biocide effectiveness. Biofilms formed by two different microbial consortia from different oilfield production facilities were used to assess whether a similar response to nutrient level can be expected from different microbial communities. Assessing the impact of dynamic flow on biofilm activity and the resulting effectiveness of biocide treatments is important in order to understand the risk of MIC through oil production facilities, which contain piping sections exposed to continuous operational flow (nutrient replenishment) and sections that remain stagnant for long periods of time.

The results of this investigation demonstrated that biofilm morphology, thickness, biomass, activity, and composition are determined by the availability of nutrients in the environment. It was shown that both microbial consortia produced thinner biofilms with lower biomass content under batch conditions compared to biofilms formed under continuous replenishment of nutrients (Fig. 1). Less-dense biofilms observed in batch reactors are the result of exposing the microorganisms to an environment limited in essential nutrients necessary for cell growth. Moreover, a relationship between nutrient level and microbial corrosivity was found; nutrient replenishment resulted in more corrosive conditions and the formation of more corrosion products, which contributed to biofilm thickness.

ATP concentration in biofilms was significantly affected by nutrient availability; replenishment led to an increase in microbial activity and viability. However, AEC measurements carried out to determine the physiological status of biofilms indicated that all biofilm communities, regardless of the nutrient conditions, were under some level of stress. In batch reactors, stress can be directly associated with a lack of nutrients. However, for reactors operating under continuous replenishment, stress was inferred to be associated with limited diffusion of nutrients and waste accumulation due to biofilm thickness. CLSM analysis (Fig. 2) showed that such biofilms had a high concentration of dead cells in areas close to the metal surface, indicating that the cells at the bottom layers of biofilms corresponded to the stressed portion of the biofilms. It has been reported that cells in the deepest areas of the biofilm are exposed to more nutrient-depleted conditions compared to upper layers of the biofilm due to the diffusion barrier generated by EPS and other biofilm components (15, 28), as well as the consumption of nutrient during the metabolic activities carry out by cells at the periphery of the biofilm (29). EPS-like structures that could have generated this diffusion barrier were observed more abundantly in the metal surfaces of coupons exposed to both consortia under continuous replenishment (Fig. 1). Hence, our results support the view that a significant portion of the biofilms would experience nutrient limitation regardless of the availability of nutrients in the bulk solution.

Analysis of microbial composition and structure of biofilms showed dissimilarities in the total and active microbial communities for each microbial consortium and at different nutrient conditions. RNA-based sequencing showed that despite the limited nutrients available in the batch reactors, both consortia had microbial species that were metabolically active in the biofilms. Of particular interest is the detection of active methanogens such as *Methanobacterium* and *Methanoculleus* only in the batch reactor of consortium 1. Methanogenic species have been shown to display metabolic capabilities, including extracellular electron transfer and interspecies electron transfer under starvation conditions (30, 31). The lower abundance of these methanogenic species in biofilms formed in continuous flow reactors can be related to an inhibition by the sulfate-reducing bacterium *Desulfovibrio*, one of the dominant genera in these biofilms. Methanogens and sulfate reducers are known to compete for energy sources, with sulfate-reducing microbes outcompeting the methanogens in sulfate-rich environments (32). Like methanogens, the genus *Thioalbus* was a dominant microorganism only detected in biofilms formed in batch conditions of consortium 1. *Thioalbus* are chemolithoautotrophic microorganisms that can use inorganic compounds such as sulfur or thiosulphate to obtain energy and bicarbonate as a carbon source (33); these metabolic capabilities could explain their dominance in the batch reactor. Conversely, fermenting microorganisms showed an opposite pattern; the genera *Acetomicrobium*, *Petrotoga*, *Anaerophaga*, and *Thermovirga* increased their abundances in the continuously replenished reactors, which indicated that these populations were favored by the availability of nutrients, most likely organic sources needed to conduct fermentation. Substantial differences observed in the beta diversity of active communities in biofilms (Fig. 5), which were based on nutrient availability pointed out an expected shift in biofilm community composition along oil production facilities in response to flow regime.

### Influence of nutrient level on the severity of microbial corrosion.

Experimental results demonstrated that MIC was directly correlated with nutrient availability. Biofilms with a permanent flow of nutrients triggered higher pitting rates compared to biofilms with a nutrient-limited environment. Same corrosion behavior was observed for the two microbial consortia, which provided strong support to the findings of this investigation. These results are consistent with previous studies with single-species biofilms of Desulfovibrio desulfuricans (34). The authors of that study found that *D. desulfuricans* in nutrient-replenished reactors decreased the polarization resistance and accelerated corrosion rates to a greater extent than did the microorganisms in batch reactors. Similarly, Javed and coworkers (35) evaluated the difference in the corrosivity of Escherichia coli between nutrient-replenished and static batch reactors, finding that culture medium replenishment produced an increase in microbial corrosion rates. The authors of both of these studies attributed the differences in the MIC rates to the higher number of bacteria present in the most corrosive scenario. Although the number of microorganisms has been demonstrated to influence the severity of MIC (36), we attribute the differences in the microbial corrosivity to the higher number of cells in an active state, measured as the ATP concentration, under continuous replenishment of nutrients. Biofilms in continuously replenished reactors exhibited 28 times (consortium 1) and 129 times (consortium 2) more ATP than biofilms in batch mode reactors. Moreover, the severity of the corrosion was correlated with the ATP concentration; the highest pitting rate and localized corrosion were observed in the coupons exposed to the most active biofilm community (consortium 2, continuous), and the lowest pitting rate was observed in the coupons exposed to the less active biofilm community (consortium 2, batch).

The impact of microbial activity on the pitting rates found in this study is supported by the data from the microbiological characterization of biofilms. For example, the microbial compositions of the active populations in consortium 2 biofilms under both nutrient conditions were similar, although at different relative abundances, indicating that differences in the corrosivity were not related to specific microbial species present but to the level of metabolic activity carried out by these active populations. Microbial consortium 2 was constituted by similar relative proportions of sulfate-reducing bacteria (74 and 62%) and sulfidogenic fermenters (24 and 34%) under both nutrient conditions, and yet the corrosion rates were markedly different. Instead, corrosion rates were more likely to be influenced by the increased production of corrosive metabolites when nutrients were more available. Nonetheless, it is important to note that even for biofilms formed under nutrient replenishment, the majority of cells in close contact with the metal surface remained under stressed conditions, which suggests a possible nutrient limitation and accumulation of waste products due to the biofilm thickness. Waste accumulation in a sulfidogenic-fermentative biofilm can lead to the generation of concentration cells of sulfides and acidic species which are corrosive to the metal. In addition, species from the genus *Desulfovibrio*, which was the predominant microorganism in all biofilms regardless of the nutrient conditions, are very versatile and can use several molecules as energy source, including metallic iron under starvation conditions (20, 37). Biofilm communities with higher metabolic states compared to the planktonic communities in a batch reactor exposed to consortium 2 suggests that microorganisms in the biofilms used the metal, corrosion products, or biofilm components for their metabolic activities. When iron oxidation is coupled to the sulfate reduction, sulfate-reducing bacteria can accelerate anodic and cathodic reactions, causing electrical MIC (EMIC) and chemical MIC (CMIC) at the same time (38). Hence, the deterioration evidenced in the carbon steel samples could have been the result of different corrosion mechanisms that took place by the activity of microorganisms attached to the metal surfaces.

Moreover, SEM-EDS analysis demonstrated the presence of at least three different types of corrosion products. Areas with a bigger concentration of Fe and S indicated the presence of iron sulfides, suggesting that MIC was likely to be driven by biogenic H_2_S production carried out by sulfate, thiosulphate, and sulfur reducing microorganisms, which were all found active in biofilms. Corroded areas in the coupons contained a greater concentration of Fe and O, indicating the presence of iron oxides and probably iron carbonates, which could be related to the activity of the fermenting microorganisms (*Petrotoga*, *Thermovirga*, and *Acetomicrobium*) (39) that can create microenvironments with acidic conditions due to the release of organic acids and CO_2_ during metabolic activities. Apart from these two different areas, coupons exposed to consortium 2 showed areas with Fe, P, and O, suggesting the presence of iron phosphides. One of the mechanisms previously related to the activity of sulfate-reducing bacteria is associated with the production of extremely corrosive phosphorus compounds through phosphate reduction (40). Phosphine is one of the reported phosphorous compounds generated during the biotic reduction of phosphate, which can lead to the production of iron phosphide as a corrosion product. Although this corrosion process is controversial, these elements were identified in the corroded areas of the metals exposed to consortium 2 that had a dominance of the *Desulfovibrio* genus in the biofilms.

The lower pitting rates evidenced in the nutrient-limited environment were attributed to low microbial activity and the lack of electron donors and carbon sources but also to the deficiency of electron acceptors that could have restricted the use of the metal as a source of electrons. Xu and Gu (20) demonstrated that a carbon source reduction of 100% reduced carbon steel corrosion rates caused by *Desulfovibrio*, which was attributed to the formation of a weak biofilm under these conditions. However, other studies have shown that most corrosive scenarios can be achieved when there is a total reduction of carbon sources, which allegedly triggered an electrical MIC mechanism (21). Nevertheless, such experiments were carried out for very short periods of exposure, only 7 days, which may not provide enough time to observe a reduction in the metabolic status of the microorganisms and consequent reduction in corrosion rates with time in the nutrient-depleted scenarios. In addition, these studies are typically assessed under a limitation of electron donors but not of electron acceptors, which does not represent any industry setting since equipment exposed to prolonged starvation (or stagnation) is typically depleted of all essential nutrients, including electron acceptors.

The results presented here suggest that facilities experiencing long periods of stagnation will limit metabolic activity in biofilms which will result in lower MIC rates compared to flow conditions experiencing nutrient replenishment. Reactors with a continuous flow of nutrients are the practical equivalent of piping in which produced fluids are normally flowing. The greatest localized corrosion found under these conditions suggests that MIC failures are most likely to occur when a higher level of nutrients is available unless the microbial activity is controlled by routine biocide treatments. Nevertheless, it has to be highlighted that even though batch reactors showed a reduced pitting rate compared to the continuous reactors, the pitting rates evidenced in batch reactors (0.5 and 0.33 mm year^−1^) were still categorized as severe according to the NACE standard practice SP0775 (41). Batch reactors are the equivalent of piping that is only in occasional service, such as dead legs. Lower pitting rates under these conditions indicate that MIC failures would take longer to occur than under the equivalent flowing conditions provided the flow is below the critical flow for detachment.

### Effect of nutrient replenishment on biocide effectiveness against biofilms.

Biocide treatment is the most commonly used and effective mitigation strategy to prevent MIC. However, the ability of microorganisms to respond and adapt to harsh environments (42) typically results in the emergence of tolerance and the resistance of species to a variety of antimicrobial substances (43). It has been reported that the metabolic state of microorganisms can determine their response and susceptibility to antimicrobial substances; therefore, understanding how oilfield communities respond to biocide treatment when exposed to different nutrient conditions is essential for the optimization of chemical treatments.

This investigation showed that biofilms exposed to continuous replenishment of nutrients displayed a bigger percentage of reduction in microbial activity when exposed to biocide, measured as the ATP concentration, compared to biofilms formed under batch conditions. However, the microbial log reduction and the percent reduction estimation indicated that glutaraldehyde biocide had similar efficacy in biofilm communities exposed to different nutrient conditions. Hence, a greater reduction in ATP concentration observed in the biofilms of continuous reactors was associated with a greater reduction in the number of viable microorganisms under this condition, since these were present in greater numbers before the treatment. Although differences in the susceptibility of biofilms to biocide treatment based on nutrient conditions were not observed, we show here that biocide effectiveness against biofilms (measured as the number of cells that survived the treatment) was affected by nutrient conditions. According to a common industry practice, a bacterial growth threshold of 1,000 CFU/ml, mainly measured as planktonic cells, is used to classify oil systems under control for MIC (44). Differences in the number of cells between planktonic and sessile communities have been discussed by other researchers (45); hence, microbial control in this investigation was only measured in sessile populations. None of the biofilm communities in the continuously replenished reactors was below this threshold after the mitigation treatment. In contrast, reactors under batch conditions had a cell concentration considered “under control.” This suggests that biofilm regrowth after the chemical treatment in systems with continuous replenishment of nutrients will take place more easily due to the higher number of viable cells remaining in biofilms and the availability of essential nutrients for microbial growth.

Detection of viable microorganisms in biofilm communities after treatment with glutaraldehyde at a concentration where planktonic cells showed complete inhibition highlighted the importance of studying biofilms when assessing biocide effectiveness and mitigation treatments. The increased tolerance of biofilm communities to biocide compounds has been associated with a number of properties found in biofilms (46). Among them, the diffusion barrier generated by the EPS layers can prevent biocides from reaching microorganisms at the bottom of biofilms (47). Moreover, the reaction of the antimicrobial molecules with EPS components during biocide penetration decreases the concentration of the chemical that reaches microorganisms within the biofilm, which are then exposed to sublethal concentrations of the biocide (47). Biocide sublethal concentrations can lead to the survival of exposed cells and the development of antimicrobial resistance (43). Hence, we propose that differences in the effectiveness of glutaraldehyde against biofilms formed under a continuous flow of nutrients could have been related to the presence of thicker biofilms that protected a major proportion of biofilm cells, which might not have been exposed to an inhibitory concentration of the chemical.

The effectiveness of an antimicrobial substance is also highly dependent on the type of microorganism, i.e., the genus and species and sometimes even the strain (32). The persistence of microorganisms after their contact with antimicrobial substances has been associated with the presence of resistance mechanisms that can be either intrinsic or acquired (46). Although it is not possible with the results available to confirm the presence of resistance mechanisms in the surviving species, the identification of the same active genera after biocide treatment in all reactors regardless of nutrient conditions suggests that these microorganisms are less susceptible to glutaraldehyde than other genera that showed a marked decrease in relative abundance in biofilms.

Glutaraldehyde is an electrophilic biocide that reacts with thiol and secondary amine groups in proteins present in the outer layers of bacterial cell walls, leading to cell wall damage and cytoplasmic coagulation (48). This biocide has a broad spectrum of activity against bacteria, but to our knowledge, there are no studies of its efficacy in archaea. The differences in the cell wall and cell membrane between archaea and bacteria have been used to explain the ineffectiveness of some antibiotics against archaea (49). This could also be the reason for the detection of active methanogens in the community that survived the treatment. Likewise, it has been reported that Gram-positive bacteria are less susceptible than Gram-negative bacteria to biocides similar to glutaraldehyde (50), which could explain the presence of active *Bacillus* and *Lactobacillus* in biofilms after the treatment. Specific resistance mechanisms for *Petrotoga* have not been described; however, *Petrotoga* has been detected in gas formations treated with glutaraldehyde (51). This bacterium has an outer-sheath-like structure that might provide physical characteristics that confers resistance to biocides (52). The resistance of some *Pseudomonas* species to glutaraldehyde has been related to the presence of genetic mechanisms, specifically, the expression of efflux pumps and the induction of modulators of biofilm formation (53). Efflux pumps can be induced when microorganisms are under stress conditions, which contributes to higher bacterial resistance of stressed populations to antimicrobial substances and could be the reason for the greater increase in the relative abundance of *Pseudomonas* in biofilms formed in batch reactors. Although physical characteristics have been the primary mechanisms described for glutaraldehyde resistance in bacterial biofilms (53), future studies are needed to identify whether the microorganisms that survived the treatment have resistance mechanisms that make them less susceptible to glutaraldehyde or whether a diffusion barrier prevented their contact with lethal doses of the chemical.

### Conclusions.

This investigation demonstrated that the nutrient level affects biofilm characteristics and the resulting corrosive behavior and biocide effectiveness. Microorganisms grown under nutrient replenishment conditions produced a higher sessile biomass and created thicker biofilms. The constant flow of nutrients allowed biofilms to be more active and cause greater localized corrosion. Higher corrosivity was attributed to the presence of nutrients that allowed microorganisms to remain metabolically active during the experimental period, potentially constantly producing corrosive metabolites compared to biofilms formed under nutrient-limited environment. The experimental results also demonstrated differences in the biocide effectiveness at controlling microbial populations; more microorganisms survived the glutaraldehyde treatment in the continuously replenished reactors, which was associated with thicker biofilms established under this condition. Biofilm layers can generate a diffusion barrier that prevents that biocide reaches the microorganisms at the bottom of the biofilm. Finally, the complementary DNA-/RNA-based profiling revealed changes in the total and active microbial community composition and structure of biofilms as a result of nutrient level and biocide treatment and also suggested the presence of microbial species that are less susceptible to glutaraldehyde in the oilfield consortia evaluated.

## MATERIALS AND METHODS

### Oilfield microbial consortia.

Microbial consortia used in this study were recovered from two different floating production storage and offloading (FPSO) facilities located on the Australian North West Shelf. The oilfields were selected based on their substantial differences in the produced water chemical composition, aiming to recover microorganisms living under different nutritional conditions. The main difference in the chemistry between the two produced waters was the sulfate and sulfur content, which were absent in produced water from FPSO 1 but present in considerable concentrations in water from FPSO 2. In addition, the produced water from FPSO 2 exhibited a higher concentration of essential nutrients such as phosphorus, nitrogen, and total organic carbon compared to the produced water from FPSO 1. The chemical composition of the produced water from the two FPSOs is presented in the Table S1 in the supplemental material. Cultivable microorganisms recovered from produced water in these facilities were grown in the laboratory at 40°C employing different culture media to maximize the recovery of the diverse microbial populations. The culture media targeted different populations, including sulfate- and thiosulfate-reducing bacteria, methanogenic archaea, acid-producing bacteria, iron-reducing bacteria, and iron-oxidizing bacteria, separately. The composition of the culture medium is presented in Table S2. After 28 days of incubation, culture media that exhibited positive growth were mixed in equal proportions for the establishment of one microbial consortium from each FPSO.

### Evaluation of the effect of nutrient replenishment on biofilm characteristics. (i) Sample preparation.

Carbon steel round coupons of 1.27-cm^2^ exposed surface area were used for biofilm formation. Steel used had the following elemental composition (weight %): C (0.43 to 0.5), Mn (0.6 to 0.9), Si (0.15 to 0.35), S (0.01 to 0.35), P (0 to 0.035), Cr (0 to 0.40), and Fe (balance). Samples were electrocoated with a protective epoxy (Powercron 6000CX; PPG Industrial coatings) to limit the working surface to only one side of the coupons. The working surface in each coupon was wet ground to a 600-grit finish using silicon carbide paper. The samples were then washed with Milli-Q water, degreased with acetone, washed with ethanol, and dried with nitrogen gas. Before immersion, the samples were sterilized by a 15-min exposure to UV radiation.

### (ii) Test conditions.

To determine the effect of nutrient level on biofilm characteristics, sterile coupons were fixed in reactors and exposed separately to both microbial consortia for 40 days. Four anaerobic CDC biofilm reactors (Biosurface Technologies Corporation) operating in batch or continuous mode were used for the experiments. Anaerobic conditions were maintained throughout the test by continuous sparging of a gas mixture of 20% CO_2_ and 80% N_2_. Synthetic produced water supplemented with nutrients was used as a test solution for the growth of the consortia. The test solution had the following composition: 1.4 mM CaCl_2_⋅2H_2_O, 1.5 mM MgCl_2_⋅6H_2_O, 2 mM K_2_HPO_4_, 1.7 mM KH_2_PO_4_, 410 mM NaCl, 5 mM NH_4_Cl, 59 mM NaHCO_3_, 8 mM Na_2_SO_4_, 4 mM Na_2_S_2_O_3_⋅5H_2_O, 10 mM sodium pyruvate, 30 mM sodium formate, 6 mM sodium lactate, 20 mM sodium acetate, and 10 ml/liter of vitamin solution and trace elements solution (German Type Culture Collection [DSMZ], medium 141). The pH of the test solution was adjusted to 7.3 ± 0.2 with deoxygenated NaHCO_3_ solution (100 mM). Agitation of the baffle in each reactor was set to 50 rpm to maintain a homogeneous solution throughout the test. The temperature in each reactor was set to 40 ± 1°C. Reactors were inoculated with the microbial consortia at using concentrations of 10^6^ cells/ml. All reactors were operated in a batch mode for the first 72 h to allow settlement and facilitate biofilm formation. After this period, two reactors were switched to continuous flow of fresh test solution at a rate of 0.21 ml min^−1^, which replaced 50% of the total volume (600 ml) of test solution daily. The other two reactors remained under batch mode, i.e., without nutrient replenishment, for the duration of the test.

### (iii) Microscopic examination of biofilms.

*(a) Field emission scanning electron microscopy*. Biofilm morphology was examined under a Tescan Mira-3 field emission scanning electron microscope. At the completion of immersion, coupons were gently rinsed with sterile anaerobic phosphate-buffered solution (PBS), and biofilms were fixed for 22 h with a 2.5% glutaraldehyde fixative solution containing 0.15% alcian blue. After fixation, coupons were rinsed again with PBS and dehydrated using a series of ethanol gradient solutions (30, 50, 70, 80, 90, 95, and 100%) for 10 min each. Dehydrated coupons were dried under nitrogen flow for 2 days, coated with a platinum layer (5 nm thick), and stored in a vacuum desiccator until imaging. Biofilms were visualized at an emission voltage of 5 kV.

*(b) Confocal laser scanning microscopy*. The distribution of live and dead cells within biofilms was studied using CLSM. Coupons were gently rinsed with sterile anaerobic PBS and stained using the FilmTracer Live/Dead biofilm viability kit (Invitrogen) according to the manufacturer’s instructions. Before imaging with a Nikon A1RMP confocal and multiphoton phosphorus, coupons were rinsed with sterile deionized water to remove the excess of dyes. Images were obtained with a 20× water lens objective. The dyes used stained live cells with a green-fluorescent color (SYTO 9) and dead cells with a red color (propidium iodide). The z-stacked images were analyzed using Nikon Elements AR software.

*(c) 3D optical profilometer*. 3D profiling of the biofilms and corrosion products attached to the carbon steel surfaces was reconstructed with an Alicona imaging infinite focus microscope IFM G4 3.5. The images were used to assess the distribution of the biofilms over the coupons and to estimate biofilm thickness for each condition.

### (iv) Microbial viability in biofilms via MPN enumeration.

The numbers of sulfide-producing prokaryotes (SPP) and total anaerobic microorganisms (ANA) in biofilms were determined by a culture-dependent MPN method using culture medium SPP described in Table S2. After exposure, biofilmed coupons were gently washed with sterile anaerobic PBS to remove unattached cells and immersed in Falcon tubes containing 10 ml of PBS solution. The cells were detached from the metal by sonication as described elsewhere (54). Portions (1 ml) of PBS sonicate suspension were inoculated into 9 ml of culture media, and then 10-fold serial dilutions up to 10^12^ were performed in triplicate for the MPN estimation. Serial dilution vials were incubated at 40°C for 28 days, and positive growth was determined by visual inspection of changes in the turbidity and the color of the culture media. Positive vials were confirmed by phase-contrast microscopy (Nikon Eclipse Ci-L). The microbial concentration was determined using the three-tube standard table for MPN (55). The number of planktonic cells in the bulk fluid of the reactors was determined by the MPN method described above; in this case, 1 ml of the bulk test solution was used for each serial dilution. The detection limit of the MPN method was 3 MPN cm^−2^ for sessile cells and 0.3 MPN ml^−1^ for planktonic cells.

### (v) Adenylate energy charge estimation.

ATP, ADP, and AMP play an important role in the energy metabolism of all living cells. These adenosine nucleotides are linked to a chain of three, two, or one phosphate groups, respectively. The hydrolysis of the bonds between phosphate groups in these molecules provides a large amount of free energy to the microorganisms that use it to develop other cellular processes (56). The ATP content of the cell varies depending on its level of activity; actively growing cells have a higher ATP content than stressed cells. Hence, ATP concentration has been used to determine the viable biomass (57); however, the ratio of ATP, ADP, and AMP concentrations is thermodynamically more important than the absolute concentration of ATP for identifying the physiological and nutritional status of microorganisms (58). This ratio was defined as adenylate energy charge (AEC) by Atkinson and Walton (59) and corresponds to the degree to which the adenylate system is charged with donatable phosphate groups (ranging between 0 when it is all AMP and 1 when it is all ATP) (60). It is generally accepted that AEC values of >0.75 correspond to actively growing microorganisms (i.e., no stress involved), values between 0.5 and 0.75 correspond to microorganisms in a stationary growth phase (i.e., microorganisms partially stressed), and values below 0.5 correspond to senescent or dormant microorganisms (i.e., microorganisms severely stressed) (61).

The concentrations of ATP, ADP, and AMP in biofilms were determined by luminescence after reaction with luciferin-luciferase using the AXP assay and the Quench-Gone Organic Modified (QGO–M) test kits (Luminultra Technologies, Ltd.). All assays were performed according to the manufacturer’s instructions. Coupons were gently rinsed with PBS and then immersed in a Falcon tube containing 10 ml of PBS solution. Coupons were then vortexed at full speed for 10 s and sonicated for 2 min to help with cell detachment. PBS containing detached cells (sessile cells) was processed with the QGO–M kit. The ATP, ADP+ATP, and AMP+ATP concentrations were determined by measuring luminescence with a PhotonMaster luminometer (Luminultra Technologies, Ltd.) before and after biocide treatment. The AEC in biofilms was calculated according to the following formula: AEC = (ATP + 0.5ADP)/(ATP + ADP + AMP). The AEC of planktonic cells in each reactor was determined following the same method described before; in this case, 10 ml of the bulk test solution was processed with the QGO–M kit.

### (vi) Microbial community composition.

The microbial composition of the total and active communities in the biofilms was determined by the 16S rRNA gene and transcript sequencing. This complementary methodological approach allows identifying the relative abundances of all microorganism in the biofilm by analysis of the total DNA and the relative abundance of active microorganisms in the biofilm at the time of sampling by analysis of the RNA molecule (27). For this, six coupons from each reactor were collected, rinsed with PBS, and immersed in sterile 2:1 RNAprotect bacteria reagent (Qiagen)–PBS-Tween 20 (0.1% wt/vol). Sessile cells were detached by sonication as described elsewhere (54). Cells were harvested by centrifugation at 15,000 × *g* for 5 min at 4°C and preserved at –80°C until nucleic acid extraction.

*(a) Nucleic acid extraction and cDNA synthesis*. Extraction of the DNA and RNA molecules was performed with DNeasy PowerBiofilm and RNeasy PowerBiofilm kits (Qiagen), respectively, as recommended by the manufacturer, with minor modifications. Cell lysis was performed in a FastPrep-24 5G instrument at 6.5 m/s for 40 s, and nucleic acids were eluted with 60 μl of nuclease-free water. The DNA and RNA concentrations were quantified fluorometrically with the Qubit dsDNA and RNA HS assay kits (Life Technologies). Subsequently, total RNA was treated with a Turbo DNA-free kit (Invitrogen) to remove the remaining DNA. To verify the complete removal of DNA, a PCR targeting the 16S rRNA gene was performed using 27f and 1492r primers, described elsewhere (62). RNA was purified by using an RNeasy MinElute cleanup kit (Qiagen) and converted to cDNA by using a SuperScript IV first-strand synthesis system (Invitrogen).

*(b) Library preparation and sequencing*. Library preparation and sequencing were performed by the Australian Genome Research Facility (AGRF). Briefly, DNA and cDNA were used to amplify the V3 and V4 regions of the 16S rRNA gene by PCR. Amplicons were generated by using the primers 341F (5′-CCTAYGGGRBGCASCAG-3′) and 806R (5′-GGACTACNNGGGTATCTAAT-3′) (63). PCR was performed in Applied Biosystem 384 Veriti system following standard PCR protocol in 50-μl reactions for 29 cycles with denaturation at 94°C for 30 s, annealing at 50°C for 60 s, and extension at 72°C for 60 s. PCR products were indexed in a second PCR using TaKaRa *Taq* DNA polymerase (Clontech). Reactions were purified and paired end sequenced on an Illumina MiSeq instrument with a V3 (600 cycles) kit (Illumina).

*(c) Bioinformatics analysis*. The Qiime2 (version 2019.4) (64) software pipeline was used for data analysis. Reads were demultiplexed and assigned to respective samples according to their barcodes by the AGRF. Next, the q2-dada2 (65) plugin was implemented for quality control, trimming, dereplication, and chimera removal. Based on the demux-summary.qzv file, forward reads were truncated at 280 bases and reverse reads were truncated at 220 bases. Afterwards, low-frequency sequences were removed by using the q2-feature-table plugin. Representative sequences were taxonomically classified using the q2-feature-classifier (66) and q2-classify-consensus-blast (67) plugins against the SILVA database version 132 (68). The alpha and beta diversity metrics were calculated using diversity q2-core-metrics-phylogenetic and q2-alpha-diversity plugins. To visualize the multivariate dispersion of the community composition under each condition, a nonmetric multidimensional scaling (NMDS) analysis was conducted employing R (v3.4.3) (69). NMDS was performed based on the weighted UniFrac distance (70); lines for joining samples collected in the same reactor were projected onto the ordination, utilizing the function ordiellipse.

### Evaluation of the effect of nutrient replenishment on microbial corrosion. (i) Corrosion measurements and surface analysis.

Corrosion analysis was carried out on three coupons from each reactor after 40 days of biofilm growth. Corrosion products and biofilms were removed from the surface using Clarke’s solution, as described in the ASTM G1 standard (71). Corrosion rates were determined by the gravimetric technique that considers the weight loss and surface area of the metal samples (71). Surface profilometry analysis, including measurements of pit depth, was conducted using a 3D optical profilometer (Alicona imaging infinite focus microscope IFM G4 3.5). The average pit depth in each condition was obtained from the 10 deepest pits measured in each coupon. The pitting rate was calculated using the maximum pit depth found in each condition, as described in the NACE SP0775 standard practice (41). To discriminate microbial corrosion from abiotic corrosion, an additional reactor was set up and maintained under sterile conditions and surface profilometry analysis performed to obtain baseline corrosion in the absence of microorganisms.

### (ii) Cross-sectional analysis of corrosion products.

Cross-sectional imaging and elemental analysis of the corrosion products and biofilms were done by scanning electron microscopy coupled with energy-dispersive X-ray spectroscopy (SEM-EDS). One coupon was extracted from each reactor on day 40 and immediately placed in a glass cell for drying under nitrogen flow for 5 days. Subsequently, coupons were mounted in Epofix resin and one of the sides was dry polished to reveal the cross-sectional profile. Samples were coated with a platinum layer (5 nm thick), and surface analysis was performed using a Zeiss Neon high-resolution scanning electron microscope. Images were collected using 20 kV and the backscatter detector. Aztec 3.0 software (Oxford Instruments NanoAnalysis) was used for the data analysis.

### Evaluation of the effect of nutrient replenishment on biocide effectiveness. (i) MIC.

Glutaraldehyde solution grade I (25% in water) was used as the antimicrobial substance for the evaluation of biocide susceptibility in biofilms formed on carbon steel. To select the doses for the chemical treatment, an MIC test in planktonic cells was performed. Glass vials containing 50 ml of synthetic produced water (described above under “Test conditions”) were supplemented with different concentrations of glutaraldehyde (0, 50, 100, 200, 400, 1,000, 2,000, and 2,500 ppm) and were inoculated with both microbial consortia at a concentration of 10^6^ cells/ml. Glass vials were incubated at 40°C for 28 days, and then microbial cells were enumerated using a Neubauer counting chamber and a phase-contrast microscope (Nikon Eclipse Ci-L) at ×100 magnification. Complete microbial inhibition was only observed in the vials containing a concentration of 2,500 ppm of glutaraldehyde (Table S3). All analyses were performed in triplicate.

### (ii) Biocide effectiveness against biofilms on carbon steel.

Biofilms grown for a period of 40 days under the conditions mentioned previously were exposed to 2,500 ppm of glutaraldehyde biocide. For this, the synthetic produced water used as a test solution for microbial growth was washed out from reactors and replaced with sterile anaerobic PBS to avoid biocide degradation upon contact with planktonic cells and metabolic products in the test solution and also to the prevent growth of biofilm cells during biocide exposure. A concentration of 2,500 ppm of glutaraldehyde in PBS was added to each reactor operating at 40°C for a total of 4 h of contact time. After this period, biocide-treated biofilms were analyzed for viability, metabolic activity, and microbial composition, as detailed above. Cell enumeration was used to evaluate the efficacy of the treatment under each condition. The log_10_ reduction and the percent reduction of microbial cells were calculated according to the ASTM standard E2315 (72).

### Statistical analysis.

Statistical differences in the mean concentration of adenosine nucleotides found in biofilms and pit depths found in the coupons exposed to different nutrient conditions were analyzed by using PAST (v3) (73) software. The results of statistical tests were considered significant if the *P* value was *≤*0.05. The statistical analyses implemented depended on the normality of the data in each variable. The homogeneity of variance was determined by the Shapiro-Wilk test (74). To test differences in variables with normal distribution, analysis of variance, followed by Tukey’s multiple comparisons (75), was implemented. For variables with a nonnormal distribution, the Kruskal-Wallis test (76) was applied.

### Data availability.

The 16S rRNA sequences were deposited in the National Center for Biotechnology Information Sequence Read Archive under BioProject number PRJNA57919.

## Supplementary Material

Supplemental file 1
