# Supplementary material for: Nutrient Level Determines Biofilm Characteristics and Subsequent Impact on Microbial Corrosion and Biocide Effectiveness
Source: Appl Environ Microbiol. 2020 Mar 18;86(7):e02885-19. doi: 10.1128/AEM.02885-19 (PMC7082584; doi:10.1128/AEM.02885-19)
Supplement: Supplemental file 1 [file AEM.02885-19-s0001.pdf]

Supplementary material

Nutrient level determines biofilm characteristics and the subsequent impact on microbial corrosion and biocide effectiveness

Silvia J. Salgar-Chaparro[a], Katerina Lepkova[a], Thunyaluk Pojtanabuntoeng[a], Adam Darwin[b], Laura L. Machuca[a#]

[a] Curtin Corrosion Centre, WA School of Mines: Minerals, Energy and Chemical Engineering, Curtin University, Bentley, WA, Australia
[b] Woodside Energy Ltd., Perth, WA, Australia

*** Correspondence:**

Laura L. Machuca

l.machuca2@curtin.edu.au

Content

Table S1. Chemical quantitative analysis of produced water in sampled floating production storage and offloading (FPSO) facilities

Table S2. Culture media composition for microbial growth

Table S3. Minimum inhibitory concentration test results

Table S4. Most probable number (MPN) of planktonic cells exposed to different nutrient conditions

Table S5. Statistical test of the effect of nutrients on microbial activity (adenine nucleotides concentration) and pit depth.

Table S6. Adenosine nucleotides concentration and adenylate energy charge of planktonic communities grown under different nutrient conditions.

Table S7. Mean microbial community composition of biofilms grown under different nutrient conditions (%) (n = 2).

Table S8. Alpha diversity indices of biofilms grown under different nutrient conditions

Table S9. Corrosion rate calculated from the mass loss of three carbon steel coupons exposed to each condition.

Figure S1. 3D optical microscope surface image of carbon steel coupon exposed to abiotic conditions.

Figure S2. SEM images from cross-sectioned coupons exposed to different nutrients conditions. [1] resin; [2] Metal; [3] corroded area; [4] top surface layer. C1: Consortium 1, C2: Consortium 2.

Figure S3. Phosphorous and calcium maps of cross-sectioned coupons exposed to Consortium 2 under batch (A) and continuous replenishment of nutrients (B).

Table S1. Chemical quantitative analysis of produced water in sampled floating production storage and offloading (FPSO) facilities

Compound / Element	Limit of detection (mg/L)	Sample	
		FPSO 1	FPSO 2
Calcium	0.1	835	330
Iron	0.01	2.35	<0.01
Potassium	0.1	340	15
Magnesium	0.1	400	210
Manganese	0.01	0.43	0.08
Sodium	0.1	11500	8100
Nickel	0.01	0.02	<0.01
Sulphur	0.05	1	530
Zinc	0.01	<0.01	0.02
Arsenic	0.001	0.005	0.003
NOx-N	0.01	<0.01	<0.01
Nitrate-N	0.01	<0.01	<0.01
Nitrite-N	0.01	<0.01	<0.01
Ammonia-N	0.02	25.5	34
Chloride	5	19500	18000
Sulphate	1	<1	1800
Thiosulphate	2	6.8	6.9
Total Organic Carbon	1	2.5	66
Total Nitrogen	0.2	31.5	96
Total Phosphorus	0.01	0.16	7.3
Alkalinity	5	300	770
Salinity	10	36000	28000

Table S2. Culture media composition for microbial growth

Ingredients	g/L
Sulphide producing prokaryotes and methanogens (SPP)	
Ammonium chloride	0.25
Ascorbic Acid	0.1
Bacto-casitone	1
D-Glucose	1.1
Dibasic Potassium Phosphate	0.14
Iron(II) Sulphate Heptahydrate	0.5
Magnesium Sulphate Heptahydrate	4.5
Potassium Chloride	0.34
Sodium Acetate	2
Sodium Bicarbonate	5
Sodium Chloride	5
Sodium Formate	13
Sodium Lactate 50%	10 mL
Sodium Thiosulfate Pentahydrate	1.88
Yeast Extract	2
Di-Water	990 mL
Acid producing bacteria	
Beef extract	1
Bacto-casitone	10
D-Glucose	5
Sodium chloride	20
Phenol red	0.018
Di-Water	1000 mL
Iron Oxidizing bacteria	
Calcium Chloride Dihydrate	0.13
Ammonium Iron Citrate	6
Ammonium Nitrate	0.5
Dipotassium Hydrogen Orthophosphate	0.5
Magnesium Sulphate Heptahydrate	0.5
Sodium Chloride	20
Sodium Nitrate	0.5
Di-Water	1000 mL
Iron Reducing Bacteria	
Ammonium Chloride	1.5
Calcium Chloride Dihydrate	0.1
Dipotassium Hydrogen Orthophosphate	0.46
Ferric Citrate	0.703
Magnesium Chloride Hexahydrate	0.1
Magnesium Sulphate Heptahydrate	0.1
Manganese Chloride Tetrahydrate	0.005
Potassium Chloride	0.1
Sodium Acetate	0.7
Sodium Bicarbonate	2.5
Sodium Chloride	5
Sodium Molybdate Dihydrate	0.001
Yeast Extract	0.05
Di-Water	1000 mL

Table S3. Minimum inhibitory concentration test results

Glutaraldehyde Concentration (ppm)	Microbial concentration (cell/mL)
0	$(6.1 \pm 0.6) \times 10^7$
50	$(1.3 \pm 0.2) \times 10^7$
100	$(5.3 \pm 2.3) \times 10^6$
200	$(5.1 \pm 2.5) \times 10^6$
400	$(2.7 \pm 0.3) \times 10^6$
1000	$(1.5 \pm 0.2) \times 10^6$
2000	$(4.2 \pm 0.1) \times 10^5$
2500	BDL*

Errors represent standard deviation from 3 independent replicates
*BDL, below detection limit of the Neubauer counting chamber (1×10^4).

Table S4. Most probable number (MPN) of planktonic cells exposed to different nutrient conditions

Consortia	Condition	MPN SPP	95% Confidence Limit	
		Cell/mL	Lower	Higher
Consortium 1	Batch	1.10E+08	3.60E+07	4.18E+08
	Continuous	2.40E+04	4.20E+03	2.40E+06
Consortium 2	Batch	1.10E+09	1.80E+08	4.51E+11
	Continuous	3.50E+07	8.70E+06	3.29E+08

MPN determined from 3-tube serial dilutions
Abbreviations: SPP = sulphide producing prokaryotes

Table S5. Statistical test of the effect of nutrients on microbial activity (adenine nucleotides concentration) and pit depth.

**Consortia**	**Condition**	**ATP**		**ADP**		**AMP**		**Pit depth**	
		χ^2	***p*-value**	χ^2	***p*-value**	χ^2	***p*-value**	χ^2	***p*-value**
Consortium 1	Before biocide	9.4	0.012*	8.7	0.012*	7.6	0.031*	1.2	0.33
Consortium 1	After biocide	9.4	0.113	8.7	0.113	7.6	0.089	ND	ND
Consortium 2	Before biocide	9.5	0.017*	9.4	0.009**	10.4	0.041*	29.3	< 0.001***
Consortium 2	After biocide	9.5	0.089	9.4	0.141	10.4	0.041*	ND	ND

χ^2: Kruskal-Wallis chi-squared values, p-value: pairwise Dunn Test

Significance: $p \leq 0.001$ = ***, $p \leq 0.01$ = **, $p \leq 0.05$ = *, $p > 0.05$ = not significant

Abbreviation: ND = not determined

Table S6. Adenosine nucleotides concentration and adenylate energy charge of planktonic communities grown under different nutrient conditions.

Consortia	Consortium 1		Consortium 2	
**Condition**	**Batch**	**Continuous**	**Batch**	**Continuous**
ATP (pg/mL)	12,106 ± 1,616	742,608 ± 15,894	8,372 ± 1,397	568,777 ± 53,533
ADP (pg/mL)	2,830 ± 643	14,962 ± 1,789	1,096 ± 284	58,477 ± 24,961
AMP (pg/mL)	26,983 ± 4,938	443,681 ± 19,126	8,533 ± 970	396,054 ± 28,587
Total Adenylates (ng/mL)	42 ± 7.1	1,201 ± 52	18 ± 2.6	1,023 ± 82
AEC	0.32 ± 0.01	0.63 ± 0.01	0.49 ± 0.02	0.58 ± 0.01
% ATP	29 ± 1.5	63 ± 2.1	46 ± 1.5	56 ± 1.9
% ADP	7 ± 0.4	1 ± 0.7	6 ± 0.8	6 ± 2.3
% AMP	64 ± 1.1	38 ± 0.9	48 ± 2.1	39 ± 0.6

Errors represent standard deviation from 3 independent replicates

Table S7. Mean microbial community composition of biofilms grown under different nutrient conditions (%) (n = 2).

**Microorganism**	**Total (DNA)**				**Active (RNA)**			
	**Consortium 1**		**Consortium 2**		**Consortium 1**		**Consortium 2**	
	**B**	**C**	**B**	**C**	**B**	**C**	**B**	**C**
Methanobacterium (Archaea)	6.20	0.10			12.77	0.01		
Methanothermobacter (Archaea)	0.02	0.05						
Methanoculleus (Archaea)	0.19	0.20			6.23	0.48		
Micrococcus (Bacteria)					0.02			
Proteiniphilum (Bacteria)	0.07	0.32	0.02	0.10	0.16	0.14	0.23	0.55
Anaerophaga (Bacteria)	0.54	3.34	0.13	4.47	0.53	9.92	0.53	2.59
Uncultured *Prolixibacteraceae (Bacteria)*	0.30	0.02			1.71	0.02		
Bergeyella (Bacteria)					<0.01			
Bacillus (Bacteria)	0.08	0.01	13.66	18.83	0.06		0.13	0.10
Lactobacillus (Bacteria)							0.13	
Thermoanaerobacter (Bacteria)				0.14				
Uncultured *Limnochordales (Bacteria)*	0.02	0.01						
Halocella (Bacteria)				<0.01				
Paracoccus (Bacteria)					<0.01		<0.01	
Sphingomonas (Bacteria)		0.01		0.15				
Desulfocarbo (Bacteria)	0.21	0.23			7.61	0.52		
Desulfovibrio (Bacteria)	79.96	63.22	74.89	67.22	20.07	36.36	73.96	62.05
Pelobacter (Bacteria)	0.01	0.11		0.01	0.09	1.00		0.54
Geoalkalibacter (Bacteria)	0.01				0.13			
Marinobacter (Bacteria)	0.25	0.00			0.38	0.01	0.04	
Shewanella (Bacteria)							<0.01	
Pseudomonas (Bacteria)	3.70	3.59	0.21	0.48	3.09	0.22	0.90	0.77
Thioalbus (Bacteria)	1.10	0.04			36.16	0.23	0.01	0.01
Acetomicrobium (Bacteria)	4.86	17.22	7.95	1.08	7.51	28.53	16.16	4.80
Uncultured *Synergistaceae (Bacteria)*					0.03			
Thermovirga (Bacteria)	1.82	8.73	2.52	4.09	1.06	4.46	5.34	4.78
Mesotoga (Bacteria)	0.28	0.03			1.46	0.04	0.01	
Kosmotoga (Bacteria)	0.05	0.17			0.16	0.07		
Petrotoga (Bacteria)	0.33	2.60	0.61	3.42	0.76	18.00	2.56	23.81

Abbreviation: B = Batch; C = Continuous

Table S8. Alpha diversity indices of biofilms grown under different nutrient conditions

Consortia	Condition	Simpson index	Shannon index
Consortium 1	Batch	0.4 ± 0.06	1.3 ± 0.16
	Continuous	0.6 ± 0.04	1.9 ± 0.22
Consortium 2	Batch	0.5 ± 0.10	1.8 ± 0.33
	Continuous	0.6 ± 0.03	2.1 ± 0.10

Errors represent standard deviation from 2 independent replicates

Table S9. Corrosion rate calculated from the mass loss of three carbon steel coupons exposed to each condition.

	Corrosion rate (mm y^{-1})	
**Consortia**	**Batch**	**Continuous**
Consortium 1	0.04 ± 0.02	0.03 ± 0.01
Consortium 2	0.13 ± 0.01	0.41 ± 0.10

Errors represent standard deviation from 3 independent replicates

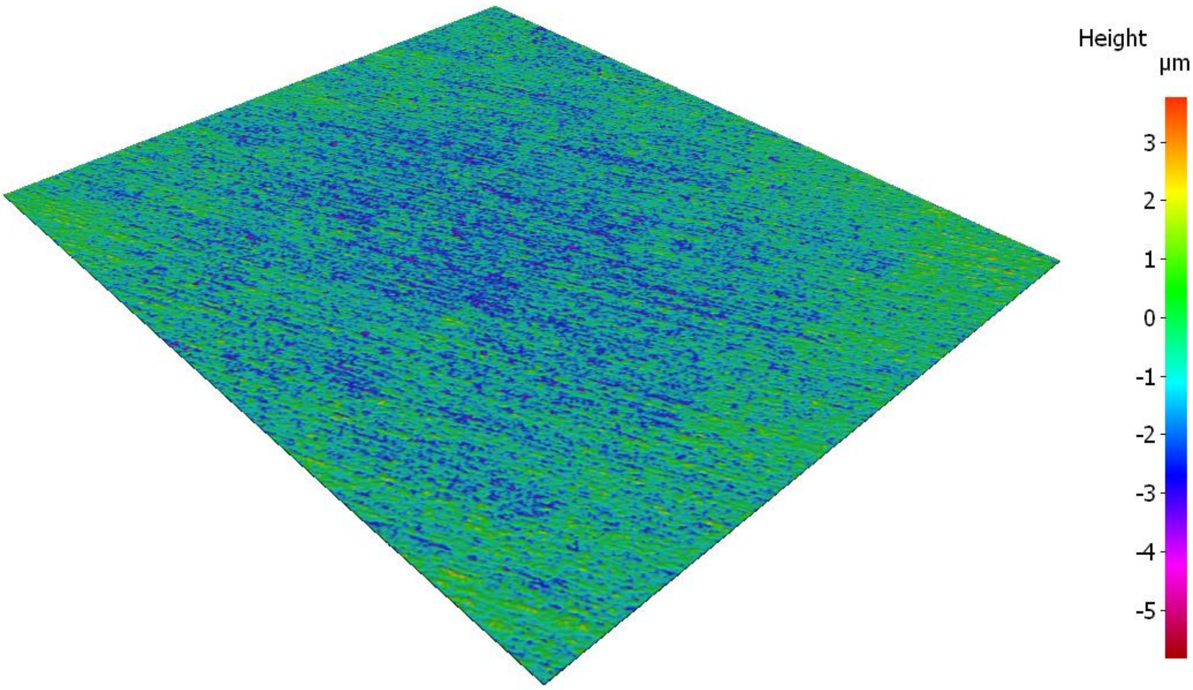

Figure S1. 3D optical microscope surface image of carbon steel coupon exposed to abiotic conditions.

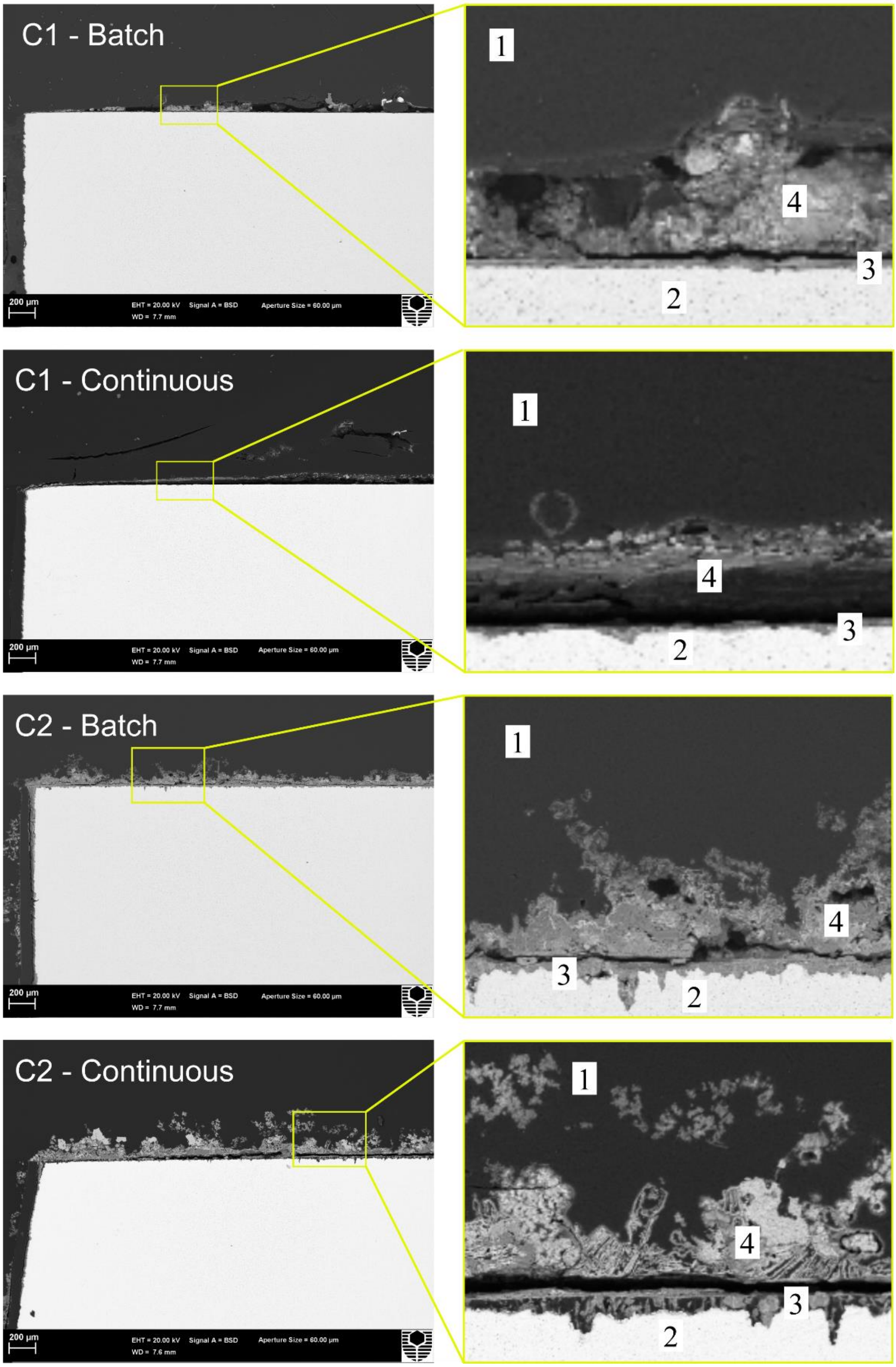

Figure S2. SEM images from cross-sectioned coupons exposed to different nutrients conditions. [1] resin; [2] Metal; [3] corroded area; [4] top surface layer. C1: Consortium 1, C2: Consortium 2.

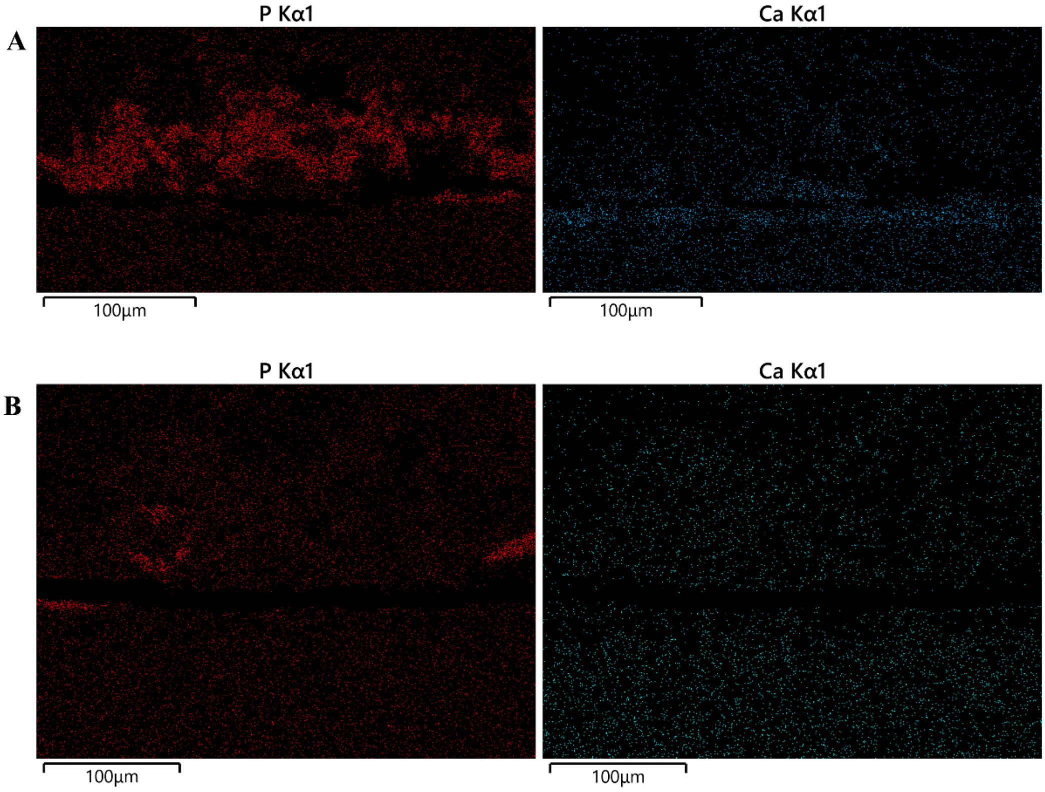

Figure S3. Phosphorous and calcium maps of cross-sectioned coupons exposed to Consortium 2 under batch (A) and continuous replenishment of nutrients (B).
